# Metabolomic Analysis of Phytochemical Compounds from Agricultural Residues of Eggplant (*Solanum melongena* L.)

**DOI:** 10.3390/molecules27207013

**Published:** 2022-10-18

**Authors:** Laura Aracely Contreras-Angulo, Aldo Moreno-Ulloa, Rommel A. Carballo-Castañeda, Josefina León-Felix, José Geovanni Romero-Quintana, Maribel Aguilar-Medina, Rosalío Ramos-Payán, J. Basilio Heredia

**Affiliations:** 1 Centro de Investigación en Alimentación y Desarrollo A.C. Carretera a Eldorado Km 5.5, Col. Campo el Diez, Culiacán CP 80110, Sinaloa, Mexico; 2 Facultad de Biología, Posgrado en Ciencias Biológicas, Universidad Autónoma de Sinaloa, Calzada de las Américas Norte 2771, Col. Burócrata, Culiacán CP 80030, Sinaloa, Mexico; 3 MS2 Laboratory, Biomedical Innovation Department, CICESE, Ensenada CP 22860, Baja California, Mexico; 4 Specialized Laboratory in Metabolomics and Proteomics (MetPro), CICESE, Ensenada CP 22860, Baja California, Mexico; 5 Facultad de Ciencias Químico Biológicas, Universidad Autónoma de Sinaloa, Calzada de las Américas Norte 2771, Col. Burócrata, Culiacán CP 80030, Sinaloa, Mexico

**Keywords:** eggplant, residues, secondary metabolites, metabolomic

## Abstract

The eggplant is a fruit rich in natural products and produced worldwide. However, its cultivation generates a large amount of scarcely used agricultural residues with poor chemical characterization. This study aimed to identify and quantify the metabolome and determine the composition of select phytochemicals and the overall antioxidant capacity of various anatomical parts of the plant. The plant’s root, leaf, stem, and fruit were analyzed by quantitative mass spectrometry-based untargeted metabolomics and chemoinformatics, and phytochemicals were quantified by spectrophotometric analysis. Moreover, we determined the total antioxidant capacity of the distinct plant parts to infer a possible biological effect of the plant’s metabolites. Various secondary metabolites were identified as terpenes, phenolic compounds, alkaloids, and saponins, distributed throughout the plant. The leaf and fruit presented the highest concentration of phenolic compounds, flavonoids, anthocyanins, and alkaloids, accompanied by the highest antioxidant capacity. Although the stem and root showed the lowest abundance of secondary metabolites, they provided around 20% of such compounds compared with the leaf and fruit. Overall, our study improved the understanding of the eggplant metabolome and concluded that the plant is rich in secondary metabolites, some with antioxidant properties, and shows potential nutraceutical and biopharmaceutical applications.

## 1. Introduction

Globally, agriculture has increased its production due to food demand [1]. This has contributed to increased environmental pollution and waste generation. It is estimated that around 998 million tons of agricultural waste are generated worldwide each year [2,3,4]. The waste is classified as by-products from fruits and vegetables, grains and legumes, the food industry, and crop residues [4,5]. This last category includes different plant parts, such as stems, leaves, roots, and fruits, which are left in the field after harvest [2,6,7].

Eggplants (*Solanum melongena* L.) are cultivated worldwide. Belonging to the *Solanaceae* family and the *Solanum* genus, eggplant is produced mainly in tropical and subtropical regions; however, it adapts perfectly to temperate climates. Furthermore, it is considered a commodity of great commercial value because it prevails in warm and humid climates. These and other characteristics allow its world production to 55,197,878 tons, with China, India, Egypt, and Turkey being the main producers [8,9,10]. México, despite not being within the top 10 places, produced 114,446.16 tons in 2020, whereas the state of Sinaloa led with 89% [11]. However, derived from the high production and the strict quality standards for its export, both globally and statewide, a large amount of agricultural waste is generated during the eggplant harvest, and a large part of the crop is not used; thus, large amounts of waste are generated that are left in the field as agricultural residues [12,13].

The exploitation of crop residues derived from primary production, such as eggplant, represents an economic opportunity and sustainable management of agricultural residues since they are a natural source of carbohydrates, lipids, fiber, fats, proteins, pigments, minerals, vitamins, and polyphenols [5,14]. Eggplant has been recognized as an important source of polyphenols derived from secondary metabolism and produced as a defense mechanism during plant growth; within this group are compounds such as phenolic acids, flavonoids, anthocyanins, and tannins [15,16,17]. For this reason, eggplant is one of the top ten vegetables with high oxygen radical absorbance capacity [16,18,19]. In some eggplant studies, the antioxidant [20], antidiabetics [21], anti-inflammatory [22,23], antiproliferative [24], analgesic [25], antimicrobial [26,27], and anticancer properties [28,29,30] are mainly related to its secondary metabolite content. A group of researchers has developed a product from eggplant peel (generally considered waste), showing an anticancer effect on skin cancer cells [31,32]. Similarly, Horincar et al. [33] produced an alcoholic beverage that added anthocyanins from eggplant peel as a functional ingredient to provide antioxidant activity. However, those investigations were conducted primarily on eggplant fruits, leaves, and stems recently harvested or acquired in markets [34,35]. Hence, the generation of information about the content of phytochemicals obtained from agricultural residues of eggplant provides an area of opportunity, as it can be recognized as an important source of these compounds for the preparation of drugs or as functional ingredients, among other applications.

The phytochemicals of agricultural residues of eggplant can be determined by different methods, such as total metabolite assays and metabolomic analysis. Metabolomics have become an important tool in various disciplines to detect as many groups of metabolites as possible, generate the fingerprint of an organism or plant, or evaluate the effect of treatments, growth processes, development, or environmental changes. In this sense, the plant metabolomic can characterize the metabolome of the plant (metabolite pool) as a response to climate conditions of stress [36,37,38].

Therefore, this research aimed to characterize the metabolites of eggplant agricultural residues (root, leaf, stem, and fruit) to know their antioxidant potential for possible use in different industries.

## 2. Results and Discussion

### 2.1. Phytochemical Screening

**Figure 1:**
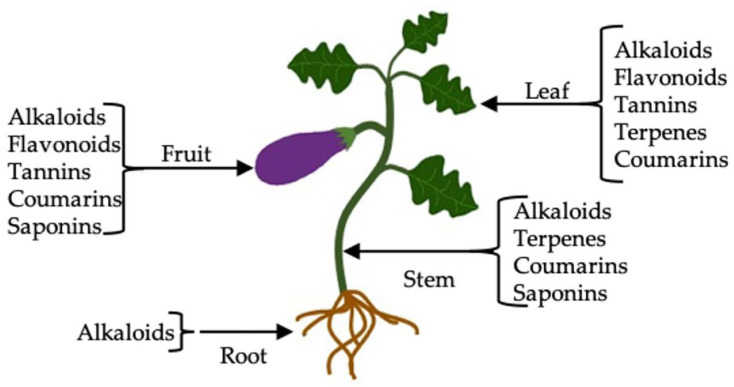
Main metabolites in eggplant organs (residues).

The results obtained from the qualitative analysis are shown in Table 1. Alkaloids were the group of secondary metabolites present in all the extracts (methanolic, hexanic, and aqueous) as well as in the different parts of the eggplant plant (Figure 1). Terpenes were observed in leaves and stems in the methanolic extract, while the group of flavonoids was present in the leaf of the extracts with the three solvents and in the fruit only in the methanolic extract. Likewise, tannins and coumarins were observed in the hexanic, methanolic, and aqueous extracts of the leaf and fruit, while saponins were in the fruit and stem extracts of the more polar solvents. The presence of terpenes in leaves may be because they fulfill the function of protection against excess light, drought, or herbivores [39]. In this sense, some phytochemicals such as alkaloids, phenolic compounds, and coumarins provide the plant an unpleasant taste that repels animals and some insects as a defense mechanism [40].

Umamageswari and Maniyar [22] reported the presence of saponins in the aqueous extract of eggplant leaves (*S. melongena* L.), which differs from our study since saponins were only found in the aqueous extract of the stem and fruit, and the methanolic extract of the fruit, perhaps due to the process used to obtain the extract. On the other hand, these same authors reported the presence of tannins and flavonoids, similar to our study. The same results were obtained by Ashrafudoulla et al. [41] in *S. melongena* leaves when evaluating aqueous extracts. Similarly, Tiwari et al. [42] carried out a phytochemical screening of *S. melongena* fruit, finding the presence of alkaloids, saponins, tannins, phenolic compounds, and flavonoids in methanolic and aqueous extracts. Most of the phytochemical screening studies in eggplant have been carried out in the fruit due to its important antioxidant contribution. In this study, the presence of different groups of phytochemicals was found in the diverse parts of the plant (root, leaf, stem, and fruit), which can be synthesized by the plant depending on the different types of stress such as lack of nutrients, dehydration, and insect attack to which it was exposed.

### 2.2. Metabolomic Analysis

**Figure 2:**
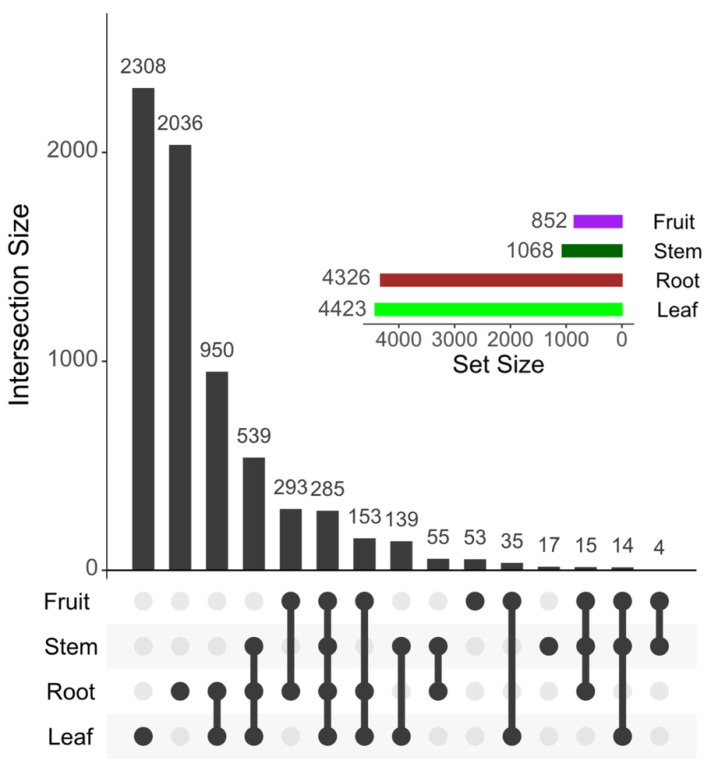
Overlapping patterns of the features detected among the eggplant anatomical sites. The 6896 aligned features (at the MS1 level) were visualized using UpSet plotting (UpSetR version 1.4.0; https://rdocumentation.org/packages/UpSetR/versions/1.4.0, accessed on 10 August 2022), wherein the bar height (intersection size) denotes the proportion of the features assigned to each unique (singular black dot or circle) or shared anatomical site (multiple linked black dots or circles). The Set Size (bars colored) denotes the total number of features detected by the anatomical site.

A total of 6896 aligned features (at the MS1 level) among all anatomical parts were extracted and filtered by MZmine analysis version 2.53 (http://mzmine.sourceforge.net/, accessed on 10 August 2022). The site with the highest number of unique features detected was the leaf (2308 features), followed by the root, fruit, and stem (Figure 2). In this sense, in some plants, the leaves are considered the arsenal of metabolites against abiotic and biotic stress [43]. The sites that shared the most features were the root and leaf, followed by the root and fruit, and root and stem. The eggplant’s metabolomic network showed many groups of metabolites; however, there are other groups of metabolites that have not been identified. Compounds such as phenol lipids, steroids, flavonoids, and cinnamic acids are predominant throughout the network.

**Figure 3:**
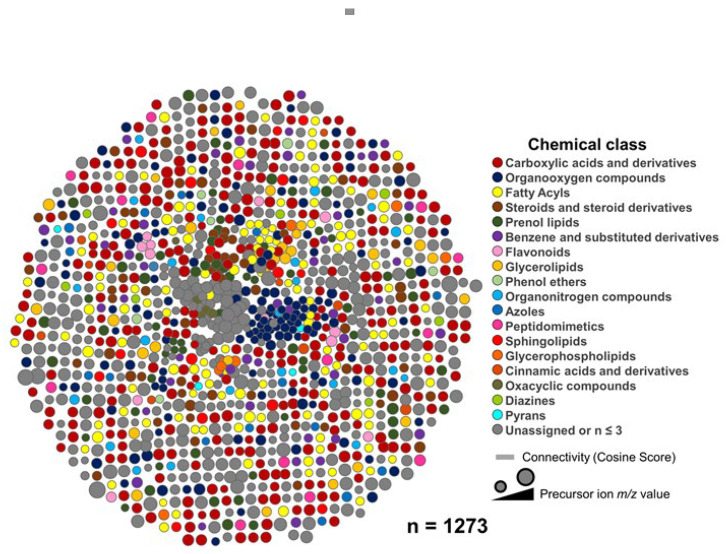
Eggplant metabolite molecular network. Chemical classes (Classyfire ontology, version 1.0 (http://classyfire.wishartlab.com, accessed on 10 August 2022) are assigned to the metabolites by the CANOPUS tool (implemented in SIRIUS Software version 4.9.12) and visualized by Cytoscape version 3.9.0 (https://cytoscape.org/, accessed on 10 August 2022). Each node represents a consensus or clustered MS2-containing feature, and the color node denotes the assigned chemical class. The *m*/*z* value of the feature is proportional to the node’s size.

We next sought to annotate the features detected in all anatomical sites at the chemical class level using the CANOPUS tool (implemented in SIRIUS Software version 4.9.12) [44], which analyzes the fragmentation spectra to predict chemical classes based on the ClassyFire ontology version 1.0 (http://classyfire.wishartlab.com, accessed on 10 August 2022) [45]. Our methodology allowed us to identify various metabolite chemical classes, as shown in Figure 3. Yet, some metabolites could not be linked to any chemical class.

**Figure 4:**
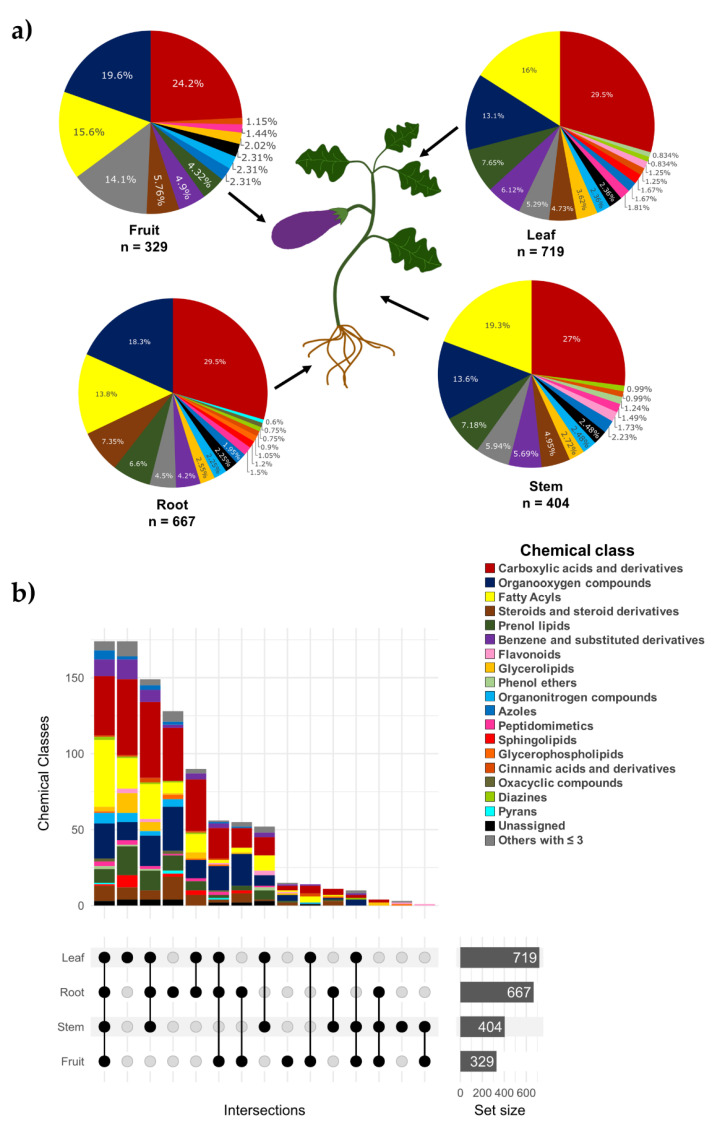
Metabolite chemical classes distributed in the different anatomical parts of eggplant residues. (**a**) Global chemical class pattern by site. (**b**) Specific overlapping pattern by site.

Globally, the most predominant classes (i.e., carboxylic acids and derivatives, fatty acyls, and organooxygen compounds) were shared among sites; however, the leaf and root presented a higher total number of chemical classes (i.e., chemical diversity) (Figure 4a,b). Aided by advanced in silico annotation (CSI: Finger ID, using a COSMIC Score >0.65) and automatic spectral matching (MSI level 2), we were able to identify 194 metabolites at the molecular structure level putatively. The complete list of the identified metabolites by the anatomical site is shown in the supplementary information (Table S1). Other studies also have reported alkaloids, terpenes, and flavonoids in *Solanum melongena* L., but they focused on the plant’s fruit [38,46,47]. For example, Hanifah et al. [48] conducted a study using an untargeted metabolomics analysis in eggplant fruit of 21 accessions, where they detected 136 and 207 peaks by LC-MS and GC-MS, respectively. Similar to our research, they identified alkaloids, terpenes, terpenoids, fatty acids, and flavonoids. However, due to the use of advanced and comprehensive annotation tools, in our study we expanded the description of the *Solanum melongena* L. metabolome and described the presence of the metabolites throughout the plant. As a result, for the first time, new chemical classes of metabolites are reported (MSI, level 3), including metabolites classified as amino acids, peptides and analogs, and carbohydrates and carbohydrates conjugates (based on the Classyfire ontology), as shown in the supplementary information (Table S1).

**Figure 5:**
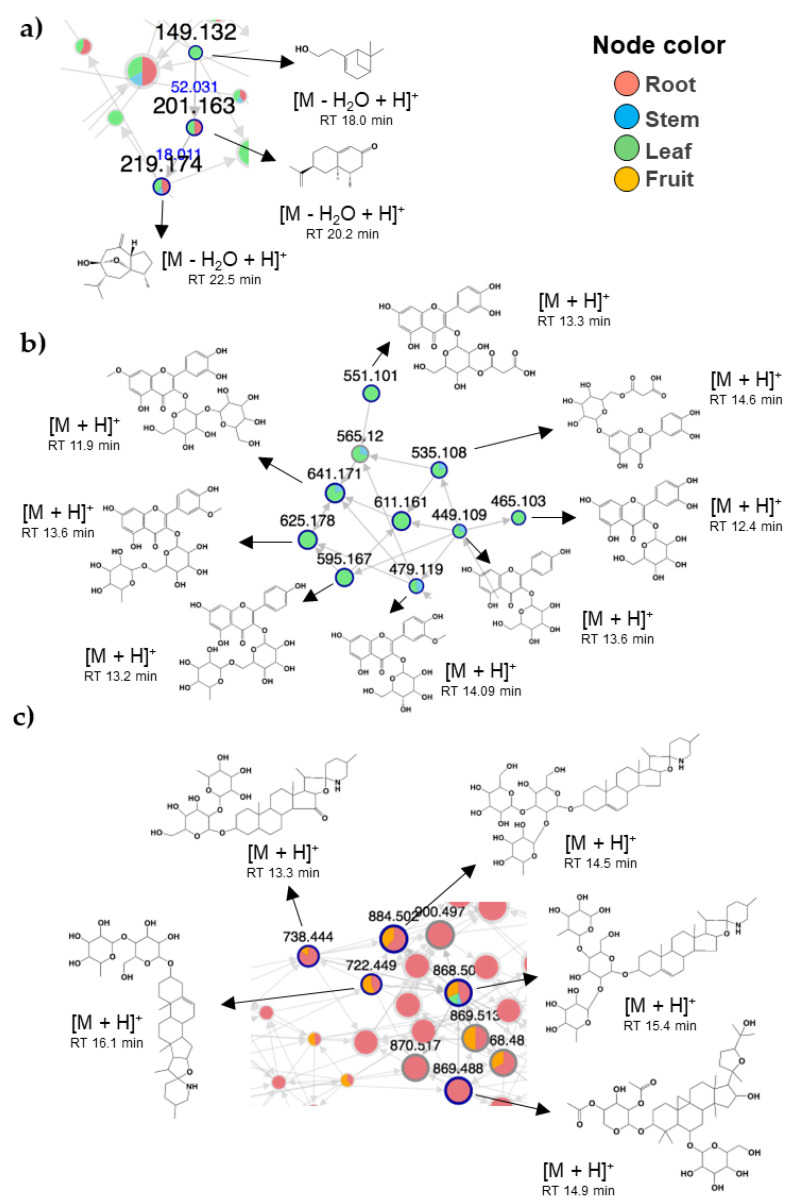
Principal group of metabolites identified in eggplant residues. Select clusters containing putatively identified metabolites and analogs. (**a**) Terpenes; (**b**) glycosides flavonoids; (**c**) saponins/alkaloids.

Due to the complexity of describing the entire set of metabolites, we focused our analysis on a select group of metabolites aided by molecular networking and automatic spectral matching as previously described by our group [49,50,51,52]. Three principal clusters (i.e., groups of metabolites with structural similarity based on MS2) containing terpenes, flavonoid glycosides, and saponins were noted (Figure 5). The distribution of those compounds varied by site. Various steroidal glycoalkaloids were only identified in the roots, including khasianine, solamargine, solasonine, and solasodine. Such metabolites have also been reported in the roots of several plants from the *Solanum* genus [53,54]. Solamargine and solasonine have been widely reported in eggplant fruit [30,55,56]; here, we noted their presence in the leaf, stem, and root, which may represent an important finding since these metabolites have been associated with beneficial effects on human health. Terpenes (monoterpenoids and sesquiterpenoids mainly) were more abundant in the leaf and root (Figure 5a), glycoside flavonoids prevailed in the leaf and stem (Figure 5a,b), mainly of the flavonol type such as quercetin 3-O-glucoside, quercetin 3-O-malonyl glucoside, cyanidin 3-O-rutinoside, rutin, luteolin 7-glucoside, delphinidin 3-rutinoside, and isorhamnetin 3-glucoside-4’-glucoside. At the same time, saponins were more abundant in the root and fruit (Figure 5c).

### 2.3. Phytochemical Content in Eggplant Agricultural Residues

#### 2.3.1. Phenolic Compounds

Eggplant is considered one of the fruits rich in phenolic acids, mostly chlorogenic acid; for this reason, the results were expressed as chlorogenic acid equivalents (CAEs) [18,57]. Data showed the highest content in the leaf, followed by the fruit, with values of 2454.93 and 2056.83 mg CAE/100 g, respectively, while in root and stem, results were <600 mg CAE/100 g (Table 2). Due to the great difference between the different parts, the significance level was high, with a value of *p* = 0.000. The higher phenolic content found in the leaf residues may be because phenolic compounds are generally located mainly in the dermal tissue of the plant rather than in the edible portion due to their protective function [58]. These results show a possible relationship with what was found in tannins and flavonoids in leaves and fruit; however, it may be that phenolic acids, such as chlorogenic acid, are the ones that provide a higher response in this test.

Plazas et al. [59] evaluated the total phenol content of 18 varieties of different shapes, sizes, and colors, finding that their content varied from 98.6 to 220 mg CAE/100 g. Likewise, Mauro et al. [17] evaluated the phenolic compounds in three varieties of ripe and overripe eggplant (*S. melongena*), finding that their values ranged between 35.4 and 105.4 mg CAE/100 g, being higher in overripe eggplant. Moreover, Lo Scalzo et al. [60] reported values from 1345 to 1578 mg CAE/100 g in fruits of different genotypes of eggplant (*S. melongena*). Similarly, Jung et al. [20] evaluated other parts of *S. melongena*, finding that fruit had the highest content of total phenolic compounds, followed by calyx, leaf, and stem. These results differ from ours, which can be related to different factors such as varieties, growing, and climatic conditions since most of those studies are carried out on eggplants obtained from supermarkets or under normal growing conditions, while in this study, samples were obtained from the field few days after the last harvest, exposing eggplant organs to more stressful situations. For this reason, to reduce environmental damage, plants may increase the production of metabolites to maintain their balance against the production of reactive oxygen species (ROS) induced by exposure to stresses [61]. According to these results obtained from phenolic compounds from eggplant plant residues, it is important to mention that they can be used in different industries, especially leaf and fruit, in the food or pharmaceutical industry mainly, since these metabolites, even at low concentrations, have the characteristic of acting as antioxidants.

#### 2.3.2. Flavonoids Content

These compounds have various beneficial pharmacological effects on health related to their antioxidant activity and can be found in all eggplant parts [62]. However, the fruit showed the highest content of total flavonoids, followed by leaves and roots with around 10%, while the stem presented the lowest values (Table 2). Likewise, the content of flavonoids found in different parts represents from 1 to 10% of the total phenolic compounds, coinciding with the range reported in the literature of 10–15% [63]. Fidrianny et al. [64] carried out a study on different parts of the plant (leaf, stem, and fruit); they found that the highest content of phenolic compounds and flavonoids was present in the leaf, followed by the fruit and stem, a behavior different from that found in this study. On the other hand, Piao et al. [65] evaluated the content of flavonoids in fruits and leaves of different accessions of brinjal from 15 countries, reporting that the content differed according to the region. Those flavonoids were 10 to 20 times higher in leaves than in fruits, concluding that the leaves are an important source of flavonoids. In addition, Boulekbache-Makhlouf et al. [66] evaluated eggplant peel, reporting in methanolic extracts 16.26 mg QE/100; these values are below what was found in this study in fruit (pulp and peel). There are differences between the diverse publications that are derived from various factors, such as the process of the samples, their origin, and variety.

#### 2.3.3. Anthocyanins

The results obtained in the total content of anthocyanins in the different parts of the eggplant plant (Table 2), showed a higher content in the fruit with 36.69 mg C_3_G/100 g, followed by the leaf with 7.34 mg C_3_G/100 g, stem (3.91 mg C_3_G/100 g), while they were not detected in roots (Table 1). The results showed a significant difference between the different parts; the significance level was high, with a value of *p* = 0.000. These data correspond to what was subjectively observed since the eggplant plant presents violet tones in its stems as well as in the petiole and nerves of the leaf (data not shown), sometimes due to the variety. However, these pigments are also expressed as a defense mechanism against stress conditions. In a study by Jiang et al. [67] the genes involved in the synthesis of anthocyanins were evaluated in different parts of the eggplant; they reported higher content in the peel, followed by stem and leaf, in root and pulp, there was no presence of anthocyanins; these results slightly differ from ours. This may be due to the type of eggplant studied by the authors, which has greater pigmentation in the stem. However, the absence of anthocyanins in the root coincides, which is explained based on the genes expression for anthocyanin synthesis is completely suppressed in the absence of light, which is why the root did not present these metabolites.

Additionally, Kadhim et al. [68] evaluated the content of anthocyanins in different parts of the eggplant plant in two physiological stages: vegetative (EV) and flowering (EF), finding values of 7.11 mg C_3_G/100 g in EF and 11.01 mg C_3_G/100 g in EV of the root; in leaf, the values were 6.20 and 6.70 mg C_3_G/100 g in EV and EF, respectively; likewise in the stem, they were 3.2 and 3.26 mg C_3_G/100 g in EV and EF, respectively. According to our results, we found similarities in leaf and stem but not in the root; this may be due to the variety or type within the same species and the stages evaluated in their study. Similarly, Jung et al. [20] carried out a study on different parts of eggplant (pulp, peel, stem, and leaf), finding that the peel had the highest content of anthocyanins, followed by the stem, leaf, and pulp, similar to our results. In general, most of the studies mentioned are not from a residue of the eggplant plant; that is, they are recently collected from a plant still in cultivation, while in this study, it is a plant with 10 days without crop management. Some studies on industrial residues have shown that they have a high content of metabolites. Lee and Wrolstad [69] obtained from waste from the blueberry industry a high content of anthocyanins with potential use as a natural source of dyes and in the nutraceutical industry.

#### 2.3.4. Tannins Total Content

The tannins found in this study are mostly hydrolyzed since when performing the analysis of the condensed tannins, values equal to zero were found (data not shown). The results demonstrated that the residue leaf of eggplant presented the highest content with values of 650.94 mg CE/100 g, followed by the fruit, stem, and root (Table 2). The differences between the different parts were highly significant, with *p* = 0.000.

In a study carried out on young and mature leaves obtained from eggplant plants grown under greenhouse conditions, tannin values were reported in a range of 2809–4840.2 mg CE/100 g, much higher than that found in the residue of the eggplant leaf, this can be due to the conditions to which they were exposed [70]. On the other hand, Al-Abdulla and Oleiwi [71] reported values of 0.008 mg CE/100 g in eggplant peel, these data are lower than that found in our study and it can be inferred that the highest content is located in the pulp since the whole fruit was analyzed. In this sense, Ashok and Upadhyaya [72] mentioned that hydrolyzable tannins in different plant species are located mainly in leaves and stems. Therefore, tannins protect plants and benefit human health, where leaf and fruit residues are the primary source of this phytochemical.

#### 2.3.5. Saponins Total Content

Saponins are abundant in the plant kingdom, particularly within the *Solanum* genus; the steroidal saponin type has been reported in different species [73,74]. The results obtained in this study are shown in Table 2, where it is observed that the highest total saponin content was found in the fruit, followed by the leaf, root, and stem with values of 43.47, 39.95, 9.84, and 8.79 mg ED/100 g. There were no significant differences in saponin content between fruit and leaf, nor between root and stem, but between fruit and leaf versus root and stem. These results do not coincide with the phytochemical screening, where the foam method reported only the presence in fruit and stem. However, it is worth mentioning that some saponins cannot produce foam [75]. In addition, Benjamin et al. [76] reported a total saponin content of 5.34 mg/100 g in eggplant (*S. melongena*) fruit, values lower than those found in the different parts of the eggplant residue plant. On the other hand, Hamzah et al. [15] reported higher values (64 mg/100 g) in ripe eggplant fruits than in residual eggplant fruit. These discrepancies can be attributed to the method used in each evaluation and to factors such as the variety, type, and origin of the studied fruits.

#### 2.3.6. Alkaloids Total Content

These metabolites are very common and biologically active within the *Solanaceae* family. Different types of these can be found, such as tropane alkaloids, glycoalkaloids, pyrrolizidine, and indole alkaloids, produced mainly as a plant defense mechanism [77]. This study shows the highest concentration of alkaloids in leaf and fruit with values of 3935 and 2689 mg EA/100 g, respectively, while in stem and root, the values were lower (Table 2). In this sense, Agoreyo et al. [78] reported in eggplant fruit round and oval type values of 1.16 and 0.99 mg/100 g, respectively. In the same way, Edeke et al. [79] reported values of 3.6 mg/100 g in the fruit of the *S. melongena* species. In a study by Kadhim et al. [68], alkaloids distributed throughout the plant (root, stem, and leaf) were found in different stages of development of the eggplant plant, similar to the results obtained in this study. Meanwhile, Mahmoud and Sadek [80] evaluated alkaloids in the leaf after the effect produced by the attack of the *Diptera Liriomyza* spp. (leaf miner) in eggplant (*S. melongena*) under greenhouse conditions, finding a variation (range from 13.1 to 20.6 g/100 g) depending on the evaluated time derived from the plant’s response to the infestation. Increasing the infestation increases the metabolites’ content; likewise, it is reduced when lowering the infestation. The data reported differ from that reported in our study, which can be due to the method used for quantification (in most studies, they are qualitative or gravimetric), as well as the very nature of the study sample and growing conditions. On the other hand, the alkaloids mainly studied in eggplant are glycoalkaloids such as solamargine and solasonine [81].

### 2.4. Antioxidant Activity

ABTS assay is one of the most sensitive antioxidant capacity methods reported that allows the evaluation of lipophilic and hydrophilic compounds, thus providing an overview of the group of compounds that can interact with the ABTS cationic radical [82]. Different concentrations were evaluated according to the plant parts residues of the eggplant. The concentrations used were from 0 to up to 4752 µg/mL to inhibit the activity of the cationic radical ABTS. The leaf was the part of the plant with the highest inhibition (IC_50_ 689.5 µg/mL), while the root, stem, and fruit showed inhibition at concentrations above 2000 µg/mL (Table 3). A study by Jo et al. [83] reports an IC_50_ of eggplant peel of approximately 1000 µg/mL, values with greater inhibitory activity of the radical concerning those found in this study in fruit (pulp and peel), comparable only to what was found in leaves. Moreover, in ten eggplant genotypes, Sharma et al. [84] reported IC_50_ values between 61.59 and 92.21 µg/mL.

DPPH radical is a synthetic compound relatively stable and reacts with antioxidants (neutralizes the radical) [85]. In the DPPH test, the capacity of the extracts of the parts of the eggplant residues to sequester free radicals is indicated, with the leaves showing the greatest anti-radical activity. The results of the linear regression to calculate the concentration necessary to inhibit the DPPH radical (100 µM) by 50% are presented in Table 3. The leaf had the greatest capacity to inhibit the DPPH radical because only 455.9 µg of the extract is required to reduce the radical to 50%. The fruit and stem need more than 2000 µg of extract, and as for the root, a concentration of 4249.9 µg of the extract is necessary to inhibit the radical.

Diatta et al. [86] reported the mean inhibitory concentration of ethanolic extracts obtained from the fruit (IC_50_ of 3.37 µg/mL) and stem (IC_50_ of 4.46 µg/mL) of eggplant, while Jung et al. [20] found IC_50_ values of 4940 µg/mL in leaves, 13,280 µg/mL in fruit (pulp and peel), and 13,130 µg/mL in the stem. In addition, Sharma et al. [84] showed IC_50_ between 74.21 and 124.58 µg/mL in eggplant fruits of different genotypes; in the same way, in a study carried out on various types of eggplant, they found IC_50_ values in a range of 433.4 to 1149.2 µg/mL [87]. These results differ from that reported in our study, possibly due to different factors such as the extraction process, variety, and parts analyzed, especially because our experiment used vegetable residues from the eggplant plant; however, these results show that there is an antioxidant effect in the residues of the eggplant plant. Likewise, according to the metabolites analyzed (Table 3), this capacity to reduce the DPPH and ABTS radicals can be related to the content of total phenols, tannins, and total alkaloids, since they present the same antioxidant behavior, as well as also due to a synergistic effect of these compounds with other antioxidants such as vitamin C, among others [88].

## 3. Materials and Methods

### 3.1. Obtaining and Processing of Agricultural Residues

American eggplants (*S. melongena* L.) were collected in June 2021, 10 days after the last harvest (open field) from a farm in the Culiacan Valley, Sinaloa, Mexico. Plants were sectioned by organ (root, leaf, stem, and fruit), washed with water, a chlorinated solution of 50 ppm of commercial chlorine, and finally with distilled water, then were frozen at −20 °C and −70 °C to be freeze-dried at −50 °C and 0.080 mbars, ground, and stored in airtight bags.

### 3.2. Phytochemical Screening

The presence/absence of secondary metabolites in residues of the different parts of the eggplant was performed through color change or precipitate formation assays such as flavonoids (Shinoda Method), phenolic compounds (Ferric chloride), alkaloids (Dragendorff, Wagner and Mayer Methods), triterpenes and steroids (Liebermann–Buchard Method), and coumarins (Barjet Method). The results were expressed as the relative presence of the metabolites, considering the following symbols: presence [+] and absence [−] [35]. The different tests were performed with the extracts obtained as follows: weighing 0.1 g of freeze-dried sample (root, stem, leaf, and fruit), and 20 mL of three solvents (hexane, methanol, and water) were added to extract the groups of metabolites present in each sample. It was mixed for 1 min, stirred at 200 rpm for 24 h at 23 °C, then centrifuged at 10,000 rpm at 4 °C for 15 min to obtain the supernatant at which the above qualitative colorimetric tests were performed.

### 3.3. Mass Spectrometry-Based Untargeted Metabolomic Analysis

**Figure 6:**
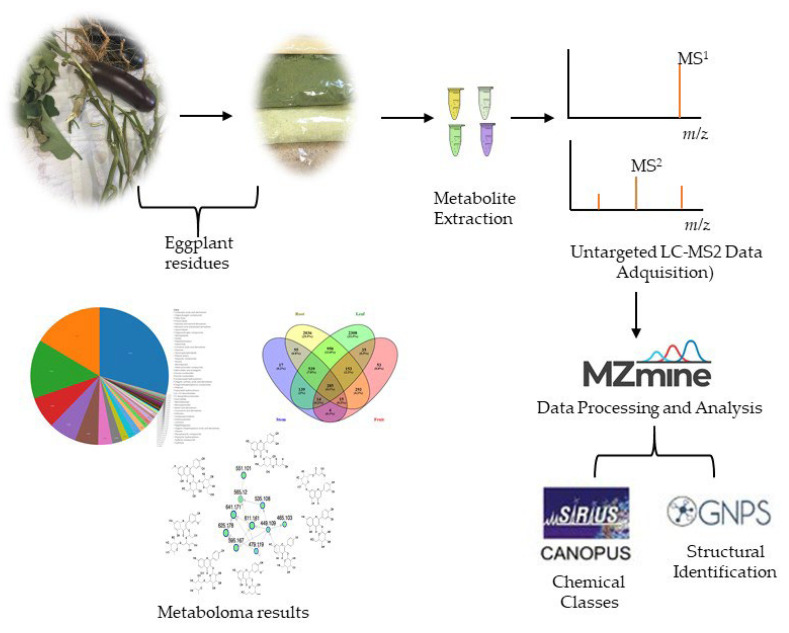
Methodological strategy of metabolomic analysis.

Figure 6 shows a schematic overview of the methodological strategy of metabolomic analysis: extraction, data acquisition, and data processing. This last step was developed with different chemoinformatic tools.

#### 3.3.1. Metabolite Extraction

A total of 5 mg of freeze-dried sample (root, stem, leaf, and fruit) was extracted with 500 µL of a mixture of methanol: ethyl acetate: acetonitrile (1:1:1) under sonication for 30 min at room temperature. Next, the samples were centrifuged at 14,000 rpm for 10 min at 4 °C. The supernatant was recovered (400 µL top layer), transferred to an Eppendorf tube, and dried with a SpeedVac system at room temperature. Finally, the dried extract was weighed, resuspended with a mixture of 80:20 (water: ACN) at a concentration of 300 ng/µL, centrifuged at 14,000 rpm for 10 min at 4 °C, and the particle-free supernatant was recovered for further analysis (content of total phenols, total flavonoids, total tannins, and antioxidant capacity).

#### 3.3.2. Untargeted LC-MS2 Data Acquisition

Sample extracts were injected (600 ng) into an Agilent 1260 Infinity LC (Agilent Technologies, Inc., Santa Clara, CA, USA). The molecules were separated through a ProtID-Chip-43 II column (C18, 43 mm, 300 Å, 5 µm particle size, equipped with a 40 nL enrichment column). The mobile phases consisted of H_2_O with 0.1% formic acid (FA) as solution A and acetonitrile (ACN) with 0.1% FA as solution B. The gradient started at 5% B, increased linearly to 20% B in 20 min, maintained at 20% B for 5 min, increased linearly to 100%, maintained at 100% B for 5 min, returned to 5% B in 1 min and then maintained at 5% B for 9 min before the next sample (to ensure column re-equilibration). The total run time was 40 min at a flow rate of 300 nL/min. Two blank samples (3 µL of mobile phases A and B at a 95:5 ratio) were run between experimental sample injections to minimize potential carryover. The eluate from the column was delivered to a 6530 Accurate-Mass Q-TOF mass spectrometer (Agilent Technologies, Inc., Santa Clara, CA, USA) via an HPLC-Chip Cube MS interface. Nanospray ionization under positive mode was employed. MS data were acquired using the following conditions: capillary voltage, 1850 V; gas temperature, 350 °C; drying gas flow, 5 L/min; skimmer voltage, 65 V; octapole RF, 750 V; fragmentor voltage, 175 V; and spectra acquisition rate, 4 spectra/s over a mass range of 110 to 2000 *m*/*z*. MS2 data conditions were: isolation window, narrow (1.3 *m*/*z*); spectra acquisition rate, 3 spectra/s; and max precursors per cycle, 5 over a mass range of 50–2000 *m*/*z*. The active exclusion option was enabled, set to 2 spectra, and released after 0.25 min. The ramped collision energy (CE) option was used with slope and offset values of 6 and 4, respectively. The instrument was externally calibrated before sample acquisition using ESI-L low mix concentration tuning mix solution (Agilent Technologies, Inc., Santa Clara, CA, USA) to ensure a mass accuracy of <5 ppm for both MS and MS2 data. Instrument performance was monitored during data acquisition by evaluating the signals of the blank sample. Samples were randomly allocated for data acquisition.

#### 3.3.3. LC-MS2 Data Processing and Analysis

The LC-MS2 datasets were analyzed using a workflow comprised of open-access software packages and online platforms and following two main steps: (1) features were extracted, aligned, and normalized using MZmine version 2.53 [89]; (2) automatic metabolite annotation or identification at the structure level [90] was performed using the Global Natural Products Social Molecular Networking web platform (GNPS, https://gnps.ucsd.edu, accessed on 10 August 2022) (Metabolomics Standards Initiative (MSI) classification level 2) [91] and in silico tools (CSI: FingerID) (MSI, level 3) [92,93]. The chemical classes of the detected metabolites (MSI, level 3) were retrieved by the CANOPUS tool [44,45] integrated within SIRIUS software version 4.9.12 (https://bio.informatik.uni-jena.de/software/sirius/, accessed on 10 August 2022). The detailed processing parameters for all the pipelines are found in supplemental experimental methods in Supplementary Materials [44,89,91,94,95,96,97,98,99].

### 3.4. Phytochemical Assays

#### 3.4.1. Methanolic Extraction

Freeze-dried samples of 0.2 g (root, stem, leaf, and fruit) were homogenized with 5 mL of 80% methanol and incubated for 24 h at 200 rpm at room temperature, after they were centrifuged at 10,000 rpm at 4 °C for 15 min, the supernatant was collected and stored at −20 °C until its use.

#### 3.4.2. Determination of Total Phenolics Compounds

The Folin–Ciocalteu reagent was used in the assay, following the methodology described by Swain and Hillis [100], with some modifications. In a 96-well plate, 10 µL of sample (methanolic extract, blank and standard), 230 µL of distilled water, and 10 µL of Folin–Ciocalteu reagent were added and incubated for 3 min; then, 25 µL of sodium carbonate (4 N) was added. The reaction mixture was incubated for 2 h and was read the absorbance at 725 nm using a Synergy HT microplate reader (Biotek Instruments, Inc., Winooski, VT, USA). The results were expressed as mg of chlorogenic acid equivalents (mg CAE)/100 g dry sample.

#### 3.4.3. Flavonoids Colorimetric Assay

The assay was performed according to the methodology described by Ghasemi et al. [101], with some modifications. An aliquot of 10 µL sample (methanolic extract, blank and standard), 250 µL of distilled water, 10 µL of aluminum chloride 10%, and 10 µL of potassium acetate 1 M were placed in a 96-well plate. The solution was incubated for 30 min, and the absorbance at 415 nm was read in a Synergy HT microplate reader (Biotek Instruments, Inc., Winooski, VT, USA). The results were reported as mg quercetin equivalents/100 mg dry sample (mg QE/100 g).

#### 3.4.4. Quantification of Total Tannins Content

It was carried out using the method described by Barman and Rai [102] and Makkar et al. [103]. First, the Folin–Ciocalteu reagent was used to quantify the total phenolic compounds (TPC) previously described but using a catechin curve (0 to 0.4 mg/mL). Subsequently, to precipitate the non-tannins, 50 mg of PVPP were weighed, and 500 µL of distilled water and 500 µL of extract were added, stirred in a vortex, and kept for 15 min at 4 °C, then centrifuged for 5 min at 3500 rpm and 4 °C. The supernatant (extract for non-tannin phenolics) was collected, and the TPC assay was performed to determine non-tannin phenolics (non-TPCs), using the catechin curve. The total tannins results were quantified using Equation (1), and expressed as mg equivalents catechin per 100 g dry sample (mg CE/100 g):

Total Tannins = TPC – NonTPC(1)

#### 3.4.5. Totals Anthocyanins Content

For this assay, 0.5 g of each freeze-dried sample with 10 mL of cold acidified ethanol (pH 1) were incubated at 200 rpm for 30 min at room temperature and centrifuged at 10,000 rpm at 4 °C for 15 min. The supernatant was collected, and an aliquot of 200 μL was placed in a 96-well plate and read at 535 nm in a Synergy HT microplate reader. The results were expressed as equivalent of cyanidin-3-O-glucoside per 100 g dry sample (mg C_3_GE/100 g) [104].

#### 3.4.6. Determination of Saponins Content

Saponins were quantified by the vanillin method, where 0.5 of each freeze-dried sample was added to 10 mL of 80% methanol and were stirred for 12 h, centrifuged at 10,000 rpm at 10 °C for 10 min, collecting the supernatant. The pellet was washed three times with 5 mL of 80% methanol, and the supernatant was recovered. A supernatant aliquot of 250 µL and 250 µL of the 8% vanillin reagent were mixed and placed in an ice bath, and 2.5 mL of 72% sulfuric acid was added; it was stirred in a vortex for 3 min, heated at 60 °C for 10 min, and cooled (ice bath). An aliquot of 200 μL was placed in a 96-well plate and read at 470 nm in a Synergy HT microplate reader. The results were reported as mg equivalents of diosgenin per 100 g dry sample (mg DE/100 g) [105].

#### 3.4.7. Totals Alkaloids Content

The method based on the alkaloids reaction with bromocresol green (BCG) was used, with some modifications [103,106]. A freeze-dried sample of 3 g was stirred with 45 mL of hexane for 4 h, and then the hexane was removed; the obtained pellet was dried. A sample of 0.2 g and 10 methanol were sonicated for 90 min and centrifuged at 10,000 rpm at 4 °C for 15 min. The supernatant was rotary-evaporated in a bath at 40 °C until dry; the extract was resuspended in 2 mL of 2N HCl and subsequently transferred to a separatory funnel and washed with 5 mL of chloroform (organic phase 1). For the aqueous phase, the pH was adjusted with NaOH 0.1 N to neutrality; to this solution, 5 mL of the solution of BCG was added to 5 mL phosphate buffer pH 4.7. For the organic phase, the mixture was vigorously shaken and placed in a separation funnel, and the organic phase (chloroform) was collected and quantified by spectrophotometry at 470 nm. The results were calculated as mg equivalents of atropine per 100 g dry sample (mg AE/100 g).

### 3.5. Antioxidant Activity

The antioxidant capacity of the methanolic extract of the freeze-dried sample was evaluated using two assays. The 2,2-diphenyl-1-picrylhydrazyl (DPPH) radical scavenging assay was performed as described by Thaipong et al. [107], at a wavelength of 540 nm, and the 2,2’-azino-bis (3-ethylbenzothiazoline-6-sulphonic acid) (ABTS) radical scavenging assay according to Karadag et al. [108], at a wavelength 734 nm. The results were expressed as the extract concentration that inhibits the radical’s activity to 50% (IC_50_).

### 3.6. Statistical Analyses

The experimental design was a completely randomized one-way ANOVA with three replications. Differences between means were evaluated using Tukey’s method for multiple comparisons and were considered significant at *p* < 0.05. Data were analyzed with MINITAB 17 software (Minitab, Inc. State College, PA, USA).

## 4. Conclusions

The metabolomic network of the residues of the eggplant plant showed more than 6000 compounds, which were found mostly in the leaf, followed by the root, stem, and fruit, with the main groups of metabolites being terpenes, flavonoids, and saponins/alkaloids. These results are consistent with what was found in the phytochemical analyses, where the leaf has the highest content of total phenols, flavonoids, hydrolyzable tannins, and alkaloids, which allows it to have the highest antioxidant capacity. In this sense, these results can indicate that the agricultural residues of the eggplant plant have antioxidant potential. Therefore, their use in several industries is possible since they present different groups of secondary metabolites, which can be economically exploited. Despite these results, it is necessary to conduct more studies of these agricultural residues in a targeted manner, that is, toward their possible pharmaceutical, cosmetic, or food use.

## Figures and Tables

**Table 1:** Phytochemical screening of eggplant agricultural residues. (+) Presence; (−) absence.

Metabolite	Assay	Solvent	Eggplant Residue			
			Root	Leaf	Stem	Fruit
**Alkaloids**	Dragendorff	Hexane	+	+	+	+
		Methanol	+	+	+	+
		Water	+	+	+	+
	Mayer	Hexane	−	+	+	−
		Methanol	−	+	+	−
		Water	−	+	+	−
	Wagner	Hexane	+	+	+	+
		Methanol	+	+	+	+
		Water	+	+	+	+
**Terpenes**	Libermann-Buchard	Hexane	−	+	−	−
		Methanol	−	+	+	−
		Water	−	−	−	−
	Salkowsky	Hexane	−	+	−	−
		Methanol	−	+	+	−
		Water	−	−	−	−
**Flavonoids**	Shinoda	Hexane	−	+	−	−
		Methanol	−	+	−	+
		Water		+	−	−
**Tannins**	Ferric Chloride	Hexane	−	+	−	+
		Methanol	−	+	−	+
		Water	−	+	−	+
**Coumarins**		Hexane	−	+	+	+
		Methanol	−	+	+	+
		Water	−	+	−	+
**Saponins**	Foam	Hexane	−	−	−	−
		Methanol	−	−	−	+
		Water	−	−	+	+

**Table 2:** Phytochemical composition of eggplant agricultural residues.

Eggplant	Phenolic Compounds	Flavonoids	Anthocyanins	Tannins	Saponins	Alkaloids
Residue	(mg CAE/100g)	(mg QE/100g)	(mg C_3_GE/100g)	(mg CE/100g)	(mg DE/100g)	(mg AE/100g)
Root	354.9 ±2 3 ^d^	23.1 ± 3 ^c^	0.0 ± 0 ^d^	56.4 ± 12 ^d^	9.8 ± 2 ^b^	653.4 ± 15 ^c^
Leaf	2454.9 ± 135 ^a^	39.3 ± 7 ^b^	7.3 ± 0.5 ^b^	650.9 ± 42 ^a^	40.0 ± 0.6 ^a^	3935.0 ± 173 ^a^
Stem	521.4 ± 37 ^c^	5.4 ± 2 ^d^	3.9 ± 0.8 ^c^	146.0 ± 4 ^c^	8.8 ± 0.6 ^b^	788.6 ± 67 ^c^
Fruit	2056.8 ± 193 ^b^	207.0 ± 10 ^a^	36.7 ± 1 ^a^	303.7 ± 5 ^b^	43.5 ± 5 ^a^	2689.5 ± 220 ^b^

^a–d^ Values with different letters in the same column are significantly different (*p* < 0.05).

**Table 3:** Antioxidant capacity of eggplant residues.

Eggplant Residue	ABTS Radical Scavenging IC_50_ (µg/mL)	DPPH Radical Scavenging IC_50_ (µg/mL)
Root	2086.0	4249.9
Leaf	689.5	455.9
Stem	2093.3	2412.3
Fruit	2626.4	2236.9

## Data Availability

The raw datasets have been deposited on the GNPS/MassIVE public repository [109] under the accession number MSV000089873. The parameters for classical molecular networking and spectral matching are available on the following link: https://gnps.ucsd.edu/ProteoSAFe/status.jsp?task=0091228b6fdd411ea3227dc61cccc0e2 (accessed on 10 August 2022).

## Supplementary Materials

The following supporting information can be downloaded at: https://www.mdpi.com/article/10.3390/molecules27207013/s1, Table S1: List of putatively annotated metabolites (SIRIUS 4.9.12) in *Solanum melongena* by location.
