# Supplementary material for: Metabolomic Analysis of Phytochemical Compounds from Agricultural Residues of Eggplant (Solanum melongena L.)
Source: Molecules. 2022 Oct 18;27(20):7013. doi: 10.3390/molecules27207013 (PMC9608517; doi:10.3390/molecules27207013)
Supplement: Supplementary file 1 [file molecules-27-07013-s001.zip › molecules-1932858-supplementary.pdf]

Table S1. List of putatively annotated metabolites (SIRIUS 4.9.12) in *Solanum melongena* L. by location.

Name	Superclass	Class	Subclass	Molecular Formula	Adduct	Theoretical mass	Experimental mass	Mass accuracy (ppm)	Retention time (min)	Spectral Match / Confidence Score	Location
2-((6-((4,5-dihydroxy-6-(hydroxymethyl)-2-((5,6a',9'-trimethyl-5-(((3,4,5-trihydroxy-6-(hydroxymethyl)tetrahydro-2H-pyran-2-yl)oxy)methyl)-1',3',4,4',5,5',6',6a',6b',7',8',8a',8b',9',11a',12',12a',12b'-octadecahydro-3H-spiro[furan-2,10'-naphtho[2',1':4,5]indeno[2,1-b]furan]-4'-yl)oxy)tetrahydro-2H-pyran-3-yl)oxy)-4,5-dihydroxy-2-(hydroxymethyl)tetrahydro-2H-pyran-3-yl)oxy)-6-methyltetrahydro-2H-pyran-3,4,5-triol	Lipids and lipid-like molecules	Steroids and steroid derivatives	Steroidal glycosides	C51H82O23	[M + H]+	1063.5319	1063.5334	1.3861	22.5587	Spectral Match	Root
Austinoneol	Organoheterocyclic compounds	Pyrans	Pyranones and derivatives	C24H30O6	[M + H]+	415.2114	415.212	1.2674	28.4702	Spectral Match	Root
Khasianine	Lipids and lipid-like molecules	Steroids and steroid derivatives	Steroidal glycosides	C39H63NO11	[M + H]+	722.4473	722.4502	4.0382	14.5986	Spectral Match	Root
2-Naphthaleneacetic acid, decahydro-8-hydroxy-4a,8-dimethyl-alpha-methylene-	Lipids and lipid-like molecules	Prenol lipids	Sesquiterpenoids	C15H24O3	[M - H2O + H]+	235.1698	235.1699	0.2299	18.243	Spectral Match	Root
Spirost-5-en-3-ol, (3beta,25R)-	Lipids and lipid-like molecules	Prenol lipids	Triterpenoids	C27H42O3	[M + H]+	415.3206	415.3172	-8.2164	14.4936	Spectral Match	Root
Solamargine	Lipids and lipid-like molecules	Steroids and steroid derivatives	Steroidal glycosides	C45H73NO15	[M + H]+	868.5052	868.506	0.8526	14.6289	Spectral Match	Root
Solasonine	Lipids and lipid-like molecules	Steroids and steroid derivatives	Steroidal glycosides	C45H73NO16	[M + H]+	884.5001	884.5034	3.6901	14.5241	Spectral Match	Root

[(2R,3R,4S,5S,6R)-4,5-dihydroxy-2-[5-hydroxy-2-(4-hydroxyphenyl)-4-oxo-7-[(2S,3R,4S,5S,6R)-3,4,5-trihydroxy-6-(hydroxymethyl)oxan-2-yl]oxychromen-3-yl]oxy-6-(hydroxymethyl)oxan-3-yl] (E)-3-(4-hydroxyphenyl)prop-2-enoate	Phenylpropanoids and polyketides	Flavonoids	Flavonoid glycosides	C36H36O18	[M + H]+	757.1974	757.2035	8.0864	15.9276	Spectral Match	Leaf
(E)-N-[2-hydroxy-2-(4-hydroxyphenyl)ethyl]-3-(4-hydroxy-3-methoxyphenyl)prop-2-enamide	Phenylpropanoids and polyketides	Cinnamic acids and derivatives	Hydroxycinnamic acids and derivatives	C18H19NO5	[M + H]+	330.1335	330.1342	2.0058	14.1932	Spectral Match	Leaf Root
4-[(3R,3aR,6S,6aR)-6-(4-hydroxy-3,5-dimethoxyphenyl)-1,3,3a,4,6,6a-hexahydrofuro[3,4-c]furan-3-yl]-2,6-dimethoxyphenol [IIN-based: Match]	Lignans, neolignanas and related compounds	Furanoid lignans	Unassigned	C22H26O8	[M + H]+	419.17	419.168	-4.6468	11.7681	Spectral Match	Leaf Root
Solasodine	Lipids and lipid-like molecules	Steroids and steroid derivatives	Steroidal alkaloids	C27H43NO2	[M + H]+	414.3366	414.3331	-8.2936	14.4397	Spectral Match	Fruit Root
(3beta,22R,25R)-26-(beta-D-Glucopyranosyloxy)-22-hydroxyfurost-5-en-3-yl 6-deoxy-alpha-L-mannopyranosyl-(1->2)-[beta-D-glucopyranosyl-(1->4)]-beta-D-glucopyranoside	Lipids and lipid-like molecules	Steroids and steroid derivatives	Steroidal glycosides	C51H84O23	[M - H2O + H]+	1047.5376	1047.5395	1.8052	17.9155	Spectral Match	Fruit Root
Pheophorbide A	Organoheterocyclic compounds	Tetrapyrroles and derivatives	Chlorins	C35H36N4O5	[M + H]+	593.2758	593.2768	1.7446	29.0621	Spectral Match	Fruit Leaf Root
Glycerol 1-stearate	Lipids and lipid-like molecules	Glycerolipids	Monoradylglycerols	C21H42O4	[M + H]+	359.3155	359.3158	0.8902	29.6387	Spectral Match	Fruit Leaf Root
Rutin	Phenylpropanoids and polyketides	Flavonoids	Flavonoid glycosides	C27H30O16	[M + H]+	611.1606	611.1612	1.0194	12.3155	Spectral Match	Stem Leaf

Linoleoyl ethanolamide	Organic nitrogen compounds	Organonitrogen compounds	Amines	C20H37NO2	[M + H]+	324.2896	324.2898	0.6176	27.717	Spectral Match	Stem Leaf
Bisabolene-1,4-endoperoxide	Organoheterocyclic compounds	Dioxanes	1,2-dioxanes	C15H24O2	[M - 2H2O + H]+	201.1643	201.1633	-5.247	17.1266	Spectral Match	Stem Leaf Root
Kaempferol-7-neohesperidoside	Phenylpropanoids and polyketides	Flavonoids	Flavonoid glycosides	C27H30O15	[M + H]+	595.1657	595.1681	4.0129	12.8166	Spectral Match	Stem Leaf Root
Jasmonic acid	Lipids and lipid-like molecules	Fatty Acyls	Lineolic acids and derivatives	C12H18O3	[M + H]+	211.1328	211.1314	-6.5013	17.4551	Spectral Match	Stem Leaf Root
(2R,3R,4S,5S,6R)-2-[[6-hydroxy-4-(4-hydroxy-3,5-dimethoxyphenyl)-3-(hydroxymethyl)-5,7-dimethoxy-1,2,3,4-tetrahydronaphthalen-2-yl]methoxy]-6-(hydroxymethyl)oxane-3,4,5-triol [IIN-based: Match]	Lignans, neolignanas and related compounds	Lignan glycosides	Unassigned	C28H38O13	[M + H3N + H]+	600.2656	600.2655	-0.2019	13.1444	Spectral Match	Stem Leaf Root
(1R,2S)-7-hydroxy-1-(4-hydroxy-3,5-dimethoxyphenyl)-2-N,3-N-bis[2-(4-hydroxyphenyl)ethyl]-6,8-dimethoxy-1,2-dihydronaphthalene-2,3-dicarboxamide [IIN-based: Match]	Lignans, neolignanas and related compounds	Aryltetralin lignans	Unassigned	C38H40N2O10	[M + H]+	685.2755	685.2742	-1.9427	18.2474	Spectral Match	Stem Leaf Root
Paprazine	Phenylpropanoids and polyketides	Cinnamic acids and derivatives	Hydroxycinnamic acids and derivatives	C17H17NO3	[M + H]+	284.128	284.1286	1.8562	15.1785	Spectral Match	Stem Leaf Root
Astragaline	Phenylpropanoids and polyketides	Flavonoids	Flavonoid glycosides	C21H20O11	[M + H]+	449.1078	449.1095	3.9363	13.7257	Spectral Match	Stem Leaf Root
17(18)-EpETE	Lipids and lipid-like molecules	Fatty Acyls	Fatty acids and conjugates	C20H30O3	[M - H2O + H]+	301.2168	301.2154	-4.5294	26.3018	Spectral Match	Stem Leaf Root
Isorhamnetin 3-O-glucoside	Phenylpropanoids and polyketides	Flavonoids	Flavonoid glycosides	C22H22O12	[M + H]+	479.1183	479.1201	3.7117	14.1702	Spectral Match	Stem Leaf Root

1,3-Dicaffeoyl-epi-quinic acid	Organic oxygen compounds	Organooxygen compounds	Alcohols and polyols	C25H24O12	[M + H]+	517.134	517.1354	2.7805	13.8533	Spectral Match	Stem Fruit Leaf
9,12,13,TriHODE	Lipids and lipid-like molecules	Fatty Acyls	Lineolic acids and derivatives	C18H32O5	[M - H2O + H]+	311.2222	311.2223	0.1477	25.3442	Spectral Match	Stem Fruit Leaf Root
LPC 18:3	Lipids and lipid-like molecules	Glycerophospholipids	Glycerophosphocholines	C26H48NO7P	[M + H]+	518.324	518.3236	-0.8726	25.7125	Spectral Match	Stem Fruit Leaf Root
LPC 18:2	Lipids and lipid-like molecules	Glycerophospholipids	Glycerophosphocholines	C26H50NO7P	[M + H]+	520.3397	520.3375	-4.252	26.1229	Spectral Match	Stem Fruit Leaf Root
(E)-3-(4-hydroxy-3-methoxyphenyl)-N-[2-(4-hydroxyphenyl)ethyl]prop-2-enamide [IIN-based: Match]	Benzenoids	Phenols	Methoxyphenols	C18H19NO4	[M + H]+	314.1386	314.139	1.3033	15.8207	Spectral Match	Stem Fruit Leaf Root
beta-D-Glucopyranoside, spirosol-5-en-3-yl O-6-deoxy-alpha-L-mannopyranosyl-(1->2)-O-[6-deoxy-alpha-L-mannopyranosyl-(1->4)]-	Lipids and lipid-like molecules	Steroids and steroid derivatives	Steroidal glycosides	C45H73NO15	[M + H]+	868.5052	868.5056	0.4412	17.783	Spectral Match	Stem Fruit Leaf Root
beta-D-Glucopyranoside, (3beta,22beta,25R)-26-(beta-D-glucopyranosyloxy)-22-hydroxyfurost-5-en-3-yl O-6-deoxy-alpha-L-mannopyranosyl-(1->2)-O-[6-deoxy-alpha-L-mannopyranosyl-(1->4)]-	Lipids and lipid-like molecules	Steroids and steroid derivatives	Steroidal glycosides	C51H84O22	[M - H2O + H]+	1031.5427	1031.5419	-0.7419	17.9545	Spectral Match	Stem Fruit Leaf Root
Carmabin B	Organic acids and derivatives	Carboxylic acids and derivatives	Amino acids, peptides, and analogues	C40H59N5O7	[M + H]+	722.4486	722.4474	-1.6987	15.1996	1	Fruit Leaf Root
2-amino-4-((7-carboxy-11-(4,5-dimethyl-4,4a,5,6,7,8,8a,9-octahydro-1H-benzo[f]indol-2-yl)-3a,6-dihydroxy-8-(4-(6-hydroxy-4-pentylcyclohex-2-en-1-yl)butyl)dodecahydro-1H-cyclopenta[10]annulen-2-yl)methyl)pyridin-1-ium	Organic acids and derivatives	Carboxylic acids and derivatives	Amino acids, peptides, and analogues	C49H75N3O5	[M + Na]+	808.5604	808.5559	-5.5809	29.2657	1	Leaf

phenacetyl KKR-acid	Organic acids and derivatives	Carboxylic acids and derivatives	Amino acids, peptides, and analogues	C26H44N8O5	[M + K]+	587.3072	587.3091	3.2063	18.1329	1	Leaf Root
[(1R,4S,5'S,6R,6'S,8R,10Z,12R,13S,14E,16E,20R,21R,24S)-6'-[(E)-but-2-en-2-yl]-24-hydroxy-21-methoxy-5',11,13,22-tetramethyl-2-oxospiro[3,7,19-trioxatetracyclo[15.6.1.14,8.020,24]pentacosa-10,14,16,22-tetraene-6,2'-oxane]-12-yl] 2-methylpropanoate	Organic acids and derivatives	Carboxylic acids and derivatives	Amino acids, peptides, and analogues	C39H56O9	[M + H3N + H]+	686.4268	686.4266	-0.2419	15.1693	0.99	Fruit
Fcoxanthin acetate	Lipids and lipid-like molecules	Glycerolipids	Triradylcglycerols	C44H60O7	[M + Na]+	723.4237	723.4298	8.4874	26.7678	0.99	Leaf
1-[N-(24-{N-[2-(acetyloxy)-3-acetamidopropyl]acetamido}tetracosa-3,6,18,21-tetraen-1-yl)acetamido]-3-acetamidopropan-2-yl acetate	Lipids and lipid-like molecules	Glycerolipids	Triradylcglycerols	C42H70N4O8	[M + Na]+	781.5091	781.5013	-9.9924	33.8738	0.99	Leaf
Corymbivillosol Acetate	Lipids and lipid-like molecules	Glycerolipids	Triradylcglycerols	C45H70O7	[M + Na]+	745.5019	745.501	-1.2649	34.0031	0.99	Stem Fruit Root
Val-Ala-His-Ser-Gly-Ala	Organic acids and derivatives	Carboxylic acids and derivatives	Amino acids, peptides, and analogues	C22H37N9O7	[M + H]+	540.2888	540.2904	2.9915	12.3812	0.99	Root
18:3,16:0 Sulfoglycolipid	Lipids and lipid-like molecules	Glycerolipids	Glycosylglycerols	C43H76O12S	[M + Na]+	839.4955	839.5003	5.7212	28.2057	0.98	Leaf
[(2S)-2-hydroxy-3-[(2R,3R,4S,5R,6R)-3,4,5-trihydroxy-6-(hydroxymethyl)oxan-2-yl]oxypropyl] (Z)-hexadec-9-enoate	Organic oxygen compounds	Organooxygen compounds	Carbohydrates and carbohydrate conjugates	C25H46O9	[M + Na]+	513.304	513.3038	-0.3237	24.3347	0.98	Stem Leaf Root
His-Arg-Lys(Me)-Val-Leu-Arg	Organic acids and derivatives	Carboxylic acids and derivatives	Amino acids, peptides, and analogues	C36H67N15O7	[M + H]+	822.542	822.5339	-9.8881	28.9573	0.98	Leaf

OxPC 34:1+1O(1Cyc)	Lipids and lipid-like molecules	Glycerophospholipids	Glycerophosphocholines	C42H80NO9P	[M + H]+	774.5643	774.5673	3.9271	28.2585	0.98	Root
[4-[(4R)-4-(2-pyrrolidin-1-ylethyl)-3,4-dihydro-2H-pyridine-1-carbonyl]-3-[4-(pyrrolidin-1-ylmethoxy)anilino]phenyl]-[4-(2-pyrrolidin-1-ylethyl)piperidin-1-yl]methanone	Benzenoids	Benzene and substituted derivatives	Benzoyl derivatives	C41H58N6O3	[M + H]+	683.4642	683.4681	5.6646	20.6357	0.98	Stem Leaf Root
Ac-Ala-Asp-Val-Ala-Asp-al	Organic acids and derivatives	Carboxylic acids and derivatives	Amino acids, peptides, and analogues	C21H33N5O10	[M + Na]+	538.2125	538.2142	3.225	16.4626	0.98	Stem Leaf Root
(2S,4S,5R)-N-[3-[3-[(2,3-dihydroxybenzoyl)amino]propyl-[(2S,4S,5R)-2-(2,3-dihydroxyphenyl)-5-methyl-1,3-oxazolidine-4-carbonyl]amino]propyl]-2-(2,3-dihydroxyphenyl)-5-methyl-1,3-oxazolidine-4-carboxamide	Organic acids and derivatives	Carboxylic acids and derivatives	Amino acids, peptides, and analogues	C35H43N5O11	[M + K]+	748.2596	748.2652	7.4145	15.7078	0.98	Stem Leaf Root
4-{7-[(5-{[3,4-dihydroxy-4-(hydroxymethyl)oxolan-2-yl]oxy}-3,4-dihydroxy-6-methyloxan-2-yl)oxy]-2,3a,10-trihydroxy-9a,11a-dimethyl-hexadecahydro-1H-cyclopenta[a]phenanthren-1-yl}-2,5-dihydrofuran-2-one	Lipids and lipid-like molecules	Steroids and steroid derivatives	Steroidal glycosides	C34H52O14	[M + Na]+	707.3255	707.3268	1.8955	10.7197	0.98	Leaf
2-[4'-(6-{3-cyclohexyl-6-methyl-4-azabicyclo[9.3.1]pentadeca-1(15),6,11,13-tetraen-9-yl}-1-hydroxyhepta-2,4,6-trien-2-yl)-3,5'-dihydroxy-8-(2-hydroxyethyl)-3-[2-(methylamino)ethyl]spiro[bicyclo[3.3.1]nonane-2,1'-cyclopentan]-6-en-9-ylidene]propanal	Lipids and lipid-like molecules	Sphingolipids	Ceramides	C49H70N2O5	[M + Na]+	789.5182	789.5147	-4.5469	29.4693	0.98	Leaf

4-Amino-N-{(1r,8r,9r,13r)-16-(4-Amino-2-Methylpyrimidin-5-Yl)-1-Benzyl-8-(Cyclohexylmethyl)-9-Hydroxy-13-[(1s)-1-Methylpropyl]-2,6,11,14-Tetraoxo-3,7,12,15-Tetraazahexadec-1-Yl}piperidine-1-Carboxamide	Lipids and lipid-like molecules	Fatty Acyls	Fatty amides	C41H64N10O6	[M + Na]+	815.4908	815.4931	2.7971	27.9432	0.98	Leaf
[2-Hydroxy-3-[3,4,5-trihydroxy-6-[[3,4,5-trihydroxy-6-(hydroxymethyl)oxan-2-yl]oxymethyl]oxan-2-yl]oxypropyl] hexadecanoate	Lipids and lipid-like molecules	Glycerolipids	Glycosylglycerols	C31H58O14	[M + H]+	655.3899	655.3948	7.5661	26.3375	0.97	Leaf Root
piplartine dimer A	Organic acids and derivatives	Carboxylic acids and derivatives	Amino acids, peptides, and analogues	C34H38N2O10	[M + Na]+	657.2424	657.2443	2.8889	16.8602	0.97	Stem Fruit Leaf Root
[(2S)-2-nonanoyloxy-3-[(2R,3S,4S,5R,6S)-3,4,5-trihydroxy-6-[[(2S,3R,4S,5R,6S)-3,4,5-trihydroxy-6-(hydroxymethyl)oxan-2-yl]oxymethyl]oxan-2-yl]oxypropyl] tetradecanoate	Organic oxygen compounds	Organooxygen compounds	Carbohydrates and carbohydrate conjugates	C38H70O15	[M + Na]+	789.4612	789.4652	5.0709	34.0133	0.97	Root
2'',3'',5''-Tri-O-acetylnebularine	Organic oxygen compounds	Organooxygen compounds	Carbohydrates and carbohydrate conjugates	C16H18N4O7	[M + H]+	379.1247	379.1215	-8.4965	14.1774	0.97	Fruit Root
N-[4-[2-[2,4-bis(2-methylbutan-2-yl)phenoxy]butanoylamino]-2-hydroxyphenyl]-2,2,3,3,4,4,4-heptafluorobutanamide	Organic acids and derivatives	Carboxylic acids and derivatives	Amino acids, peptides, and analogues	C30H37F7N2O4	[M + H]+	623.2714	623.2714	0.1513	12.775	0.97	Leaf
Diethyl ({4-[(2s,3r)-2-({[(3r,3as,6ar)-Hexahydrofuro[2,3-B]furan-3-Yloxy]carbonyl}amino)-3-Hydroxy-4-{isobutyl[(4-Methoxyphenyl)sulfonyl]amino}butyl]phenoxy}methyl)phosphonate	Organic acids and derivatives	Carboxylic acids and derivatives	Amino acids, peptides, and analogues	C33H49N2O12PS	[M + H]+	729.2816	729.2761	-7.5122	15.3511	0.97	Stem Leaf Root

Glu-Leu-Val-Ile-Ser	Organic acids and derivatives	Peptidomimetics	Depsipeptides	C25H45N5O9	[M + H3N + H]+	577.3561	577.3617	9.697	19.0387	0.97	Fruit Leaf Root
(2S)-2-[[(2S)-2-[[(2S)-2-[[(2S)-6-amino-2-[[(2S)-1-[(2S)-3-hydroxy-2-(methylamino)propanoyl]pyrrolidine-2-carbonyl]amino]hexanoyl]amino]-5-(diaminomethylideneamino)pentanoyl]amino]butanoyl]amino]propanoic acid	Organic acids and derivatives	Carboxylic acids and derivatives	Amino acids, peptides, and analogues	C28H52N10O8	[M + K]+	695.3607	695.3633	3.7839	12.5817	0.97	Stem Fruit Leaf Root
[6-[[6-[3,4-dihydroxy-2,5-bis(hydroxymethyl)oxolan-2-yl]oxy-3,4,5-trihydroxyoxan-2-yl]methoxy]-3,4,5-trihydroxyoxan-2-yl]methyl (E)-2-methylbut-2-enoate	Organic oxygen compounds	Organooxygen compounds	Carbohydrates and carbohydrate conjugates	C23H38O17	[M + H]+	587.2181	587.2192	1.7933	22.2828	0.96	Root
1-{[6-(aminomethyl)-3,4,5-trihydroxyoxan-2-yl]oxy}-3-(octadeca-9,12,15-trienoyloxy)propan-2-yl 12-methyloctadeca-9,12,15-trienoate	Phenylpropanoids and polyketides	Macrolides and analogues	Unassigned	C46H77NO9	[M + Na]+	810.5496	810.5541	5.5569	27.353	0.96	Stem Fruit Leaf Root
methyl 5-{2,5,5,8a-tetramethyl-6-[(2-methylpropanoyl)oxy]-7-[(3,4,5-trihydroxy-6-methyloxan-2-yl)oxy]-1,4,4a,5,6,7,8,8a-octahydronaphthalen-1-yl}-3-methylpentanoate	Organic oxygen compounds	Organooxygen compounds	Carbohydrates and carbohydrate conjugates	C31H52O9	[M + Na]+	591.3509	591.3541	5.4175	20.4548	0.96	Fruit Root
2-{[2-({3,4-dihydroxy-6,8-dioxabicyclo[3.2.1]octan-2-yl}oxy)-3,5-dihydroxy-6-(hydroxymethyl)oxan-4-yl]oxy}-6-methyloxane-3,4,5-triol	Nucleosides, nucleotides, and analogues	Purine nucleotides	Purine ribonucleotides	C18H30O14	[M + H]+	471.1708	471.1699	-1.8667	12.3812	0.96	Root
3-Aza-lipid X	Organic acids and derivatives	Carboxylic acids and derivatives	Amino acids, peptides,	C34H67N2O11P	[M + H]+	711.4554	711.4607	7.359	33.8738	0.96	Leaf

			and analogues								
5-oxo-delta-bilirubin	Organic acids and derivatives	Carboxylic acids and derivatives	Amino acids, peptides, and analogues	C33H34N4O7	[M + H]+	599.2499	599.2501	0.307	20.1278	0.96	Stem Leaf
Stearoyl Ara-C	Organic acids and derivatives	Carboxylic acids and derivatives	Amino acids, peptides, and analogues	C27H47N3O6	[M + H]+	510.3537	510.3557	3.9113	27.0211	0.95	Leaf Root
Samabig	Organic acids and derivatives	Peptidomimetics	Depsipeptides	C38H68N4O12	[M + K]+	811.4471	811.4455	-1.9743	16.9561	0.95	Stem Leaf Root
(3S,5S,6S,8S,9R,10S,13R,14S,15S,16R,17R)-10,13-dimethyl-17-[(E,2R,6S)-6-methyl-7-[(2S,3R,4S,5R)-3,4,5-trihydroxyoxan-2-yl]oxyhept-3-en-2-yl]-3-[(2S,3R,4S,5R)-3,4,5-trihydroxyoxan-2-yl]oxy-1,2,3,4,5,6,7,9,11,12,14,15,16,17-tetradecahydrocyclopenta[a]phenanthrene-6,8,15,16-tetrol	Lipids and lipid-like molecules	Steroids and steroid derivatives	Hydroxysteroids	C37H62O14	[M + Na]+	753.4037	753.4052	1.972	18.4243	0.95	Fruit Root
2-[6-(2-aminoethyl)-2-benzyl-5-(7-cyclohexylhept-4-en-1-yl)-5-[(methylamino)methyl]cyclohex-3-en-1-yl]-3-hydroxybutanoic acid	Organic acids and derivatives	Carboxylic acids and derivatives	Amino acids, peptides, and analogues	C34H54N2O3	[M + K]+	577.3772	577.3719	-9.0545	22.2468	0.95	Fruit Root
Archazolid A	Organic acids and derivatives	Peptidomimetics	Depsipeptides	C42H62N2O7S	[M + H]+	739.435	739.4286	-8.6418	22.2828	0.95	Root
Belofor KB	Phenylpropanoids and polyketides	Cinnamic acids and derivatives	Cinnamic acid esters	C34H30N10O8S2	[M + H]+	771.1761	771.1777	2.0051	12.848	0.94	Leaf
Cholic acid alpha-D-glucopyranosyl ester	Organic acids and derivatives	Carboxylic acids and derivatives	Amino acids, peptides, and analogues	C30H50O10	[M + H]+	571.3476	571.3474	-0.339	16.8016	0.94	Fruit Root

17-(acetylamino)-3-(acetyloxy)androstan-16-yl 5-(acetyloxy)-4-methylpentanoate	Lipids and lipid-like molecules	Prenol lipids	Terpene glycosides	C31H49NO7	[M + Na]+	570.3407	570.3399	-1.3175	16.829	0.94	Stem Fruit Leaf Root
4'''' deoxy EM-A enolether	Organic acids and derivatives	Carboxylic acids and derivatives	Amino acids, peptides, and analogues	C37H65NO11	[M + Na]+	722.4455	722.4448	-0.9512	18.3326	0.93	Fruit Leaf Root
Flavin adenine dinucleotide semiquinone radical	Nucleosides, nucleotides, and analogues	(5'->5')-dinucleotides	Unassigned	C27H34N9O15P2	[M + H]+	787.1722	787.1712	-1.1907	12.2005	0.93	Leaf
Ala-Arg-Thr-Lys-Gln-al	Organic acids and derivatives	Carboxylic acids and derivatives	Amino acids, peptides, and analogues	C24H46N10O7	[M + Na]+	609.3449	609.3497	7.9189	17.7774	0.93	Stem Leaf Root
1,3-Bis(2,4-dinitrophenyl)-1,3-diethylurea	Benzenoids	Phenol ethers	Unassigned	C17H16N6O9	[M + H]+	449.1051	449.1092	9.1417	13.1926	0.93	Stem Leaf Root
1-(9Z,12Z-octadecadienoyl)-glycero-3-phospho-(1''-myo-inositol)	Lipids and lipid-like molecules	Prenol lipids	Diterpenoids	C27H49O12P	[M + H]+	597.3034	597.3062	4.6943	25.4498	0.92	Stem Leaf Root
N-[(Benzyloxy)carbonyl]-L-Isoleucyl-N-[(1r)-1-(Carboxycarbonyl)-3,3-Difluoropropyl]-L-Leucinamide	Organic acids and derivatives	Carboxylic acids and derivatives	Amino acids, peptides, and analogues	C25H37F2N3O8	[M + Na]+	568.2446	568.2403	-7.6075	13.7041	0.92	Stem Fruit Root
Rodaplutin	Organic acids and derivatives	Carboxylic acids and derivatives	Amino acids, peptides, and analogues	C24H39N9O7	[M + H]+	566.3044	566.3086	7.3483	21.5313	0.92	Leaf
Antho-rwamide I	Organic acids and derivatives	Carboxylic acids and derivatives	Amino acids, peptides, and analogues	C31H46N10O7	[M + Na]+	693.3449	693.3471	3.1532	12.4244	0.92	Stem Fruit Leaf Root
Methionylpuromycin	Organic acids and derivatives	Carboxylic acids and derivatives	Amino acids, peptides, and analogues	C27H38N8O6S	[M + Na]+	625.2533	625.254	1.1673	18.4366	0.91	Stem Fruit Leaf Root
(2S)-6-amino-2-[[(2S)-4-amino-2-[[(2S)-2,6-diaminohexanoyl]amino]butanoyl]amino]hexanoic Acid	Benzenoids	Benzene and substituted derivatives	Unassigned	C16H34N6O4	[M + H]+	375.2714	375.2698	-4.074	28.3022	0.91	Leaf

6-methyl-3-phenylmethoxy-1-[3-(trifluoromethyl)phenyl]pyridazin-4-one	Benzenoids	Benzene and substituted derivatives	Diphenylethers	C19H15F3N2O2	[M + H]+	361.1158	361.1137	-5.6995	18.3709	0.91	Stem Fruit Leaf Root
2,4,6-Tri-(6-aminocaproic acid)-1,3,5-triazine	Organic acids and derivatives	Carboxylic acids and derivatives	Amino acids, peptides, and analogues	C21H36N6O6	[M + H]+	469.2768	469.2732	-7.7826	25.4036	0.9	Stem Leaf Root
3-hydroxy-4-[2-(3-hydroxy-4-{3-methyl-2-[3-methyl-2-(3-methylbutanamido)butanamido]butanamido}-5-phenylpentanamido)propanamido]-5-phenylpentanoic acid	Organic acids and derivatives	Carboxylic acids and derivatives	Amino acids, peptides, and analogues	C40H59N5O9	[M + H]+	754.4385	754.4379	-0.8317	12.4928	0.9	Root
Cyclo(D-alanyl-L-leucyl-L-leucyl-D-leucyl-L-valyl)	Organic acids and derivatives	Carboxylic acids and derivatives	Amino acids, peptides, and analogues	C26H47N5O5	[M + Na]+	532.3475	532.3464	-1.9985	26.3013	0.89	Stem Leaf Root
(2R)-2-[2-[[(2R,3R,4R,5S,6R)-3-acetamido-4,5-diacetyloxy-6-(acetyloxymethyl)oxan-2-yl]carbamothioylamino]ethyl-(4-phenylphenyl)sulfonylamino]-3-methylbutanoic acid	Organic acids and derivatives	Carboxylic acids and derivatives	Amino acids, peptides, and analogues	C34H44N4O12S2	[M + K]+	803.2034	803.2046	1.4301	14.872	0.89	Leaf
(4S)-4-amino-5-[[(2S,3S)-1-[[(2S)-1-[[(2S,3R)-4-[[(1S)-1-carboxybutyl]amino]-3-hydroxy-4-oxo-1-thiophen-2-ylbutan-2-yl]amino]-1-oxo-3-thiophen-2-ylpropan-2-yl]amino]-3-methyl-1-oxopentan-2-yl]amino]-5-oxopentanoic acid	Organic acids and derivatives	Peptidomimetics	Depsipeptides	C31H45N5O9S2	[M + Na]+	718.2556	718.2607	6.976	15.8555	0.89	Stem Leaf Root
[(2S)-3-(4-butoxycarbonylpiperazin-1-yl)-2-[[6-[(3S)-3-methoxypyrrolidin-1-yl]-2-phenylpyrimidine-4-carbonyl]amino]-3-oxopropyl]phosphonic acid	Organic acids and derivatives	Carboxylic acids and derivatives	Amino acids, peptides, and analogues	C28H39N6O8P	[M + Na]+	641.2465	641.2502	5.8194	19.2733	0.89	Stem Fruit Leaf Root

Solanamide A	Organic acids and derivatives	Carboxylic acids and derivatives	Amino acids, peptides, and analogues	C30H46N4O6	[M + H]+	559.3489	559.3451	-6.9204	19.0232	0.89	Stem Leaf Root
9a-(hydroxymethyl)-1-(5-hydroxyoxolan-3-yl)-11a-methyl-7-[(3,4,5-trihydroxy-6-methyloxan-2-yl)oxy]-hexadecahydro-1H-cyclopenta[a]phenanthrene-3a,5a,9,10-tetrol	Lipids and lipid-like molecules	Prenol lipids	Terpene glycosides	C29H48O12	[M + Na]+	611.3043	611.3078	5.5884	19.6101	0.89	Stem Leaf Root
2,4,12-Octadecatrienamide, N-(2-methylpropyl)-, (2E,4E,12Z)-	Lipids and lipid-like molecules	Prenol lipids	Diterpenoids	C22H39NO	[M + H]+	334.3104	334.3086	-5.1822	29.667	0.88	Leaf
O-Specific antigen, YR	Lipids and lipid-like molecules	Fatty Acyls	Fatty amides	C29H47N3O18	[M + Na]+	748.2752	748.2713	-5.307	16.1373	0.88	Stem Leaf Root
Phenylalanyl-arginyl-arginine	Organic oxygen compounds	Organooxygen compounds	Alcohols and polyols	C21H35N9O4	[M + Na]+	500.271	500.2706	-0.681	13.4261	0.88	Leaf
4-amino-2-[(2,4-diamino-5-{[3-amino-6-(aminomethyl)-3,4-dihydro-2H-pyran-2-yl]oxy}-3,6-dihydroxycyclohexyl)oxy]-5-methyloxane-3,5-diol	Lipids and lipid-like molecules	Fatty Acyls	Fatty amides	C18H35N5O8	[M + Na]+	472.2383	472.2394	2.2618	11.1929	0.88	Fruit Leaf
2-[2-[[1-[2-amino-3-(4-hydroxyphenyl)propanoyl]pyrrolidine-2-carbonyl]amino]propanoylamino]-4-methylpentanoic acid	Organic acids and derivatives	Carboxylic acids and derivatives	Amino acids, peptides, and analogues	C23H34N4O6	[M + Na]+	485.2376	485.2408	6.5067	13.7453	0.88	Leaf Root
S-Adenosyl-1,12-diamino-3-thio-9-azadodecane	Organoheterocyclic compounds	Azoles	Unassigned	C21H38N8O3S	[M + Na]+	505.2685	505.2674	-2.2079	22.9997	0.88	Leaf
4-tert-Butylcalix[5]arene	Organic acids and derivatives	Carboxylic acids and derivatives	Amino acids, peptides, and analogues	C55H70O5	[M + Na]+	833.5121	833.5154	3.9555	34.0923	0.87	Leaf Root
5-(4-ethylphenyl)-4-[hydroxy-(3-methyl-4-propan-2-yloxyphenyl)methylidene]-1-(3-morpholin-4-ylpropyl)pyrrolidine-2,3-dione	Organic acids and derivatives	Carboxylic acids and derivatives	Amino acids, peptides, and analogues	C30H38N2O5	[M + Na]+	529.2678	529.2643	-6.6256	11.6882	0.87	Root

N-[1-amino-3-(1H-indol-3-yl)-1-oxopropan-2-yl]-3-(cyclohexylcarbamoylamino)-1-(pyrazine-2-carbonyl)pyrrolidine-2-carboxamide	Organic acids and derivatives	Carboxylic acids and derivatives	Amino acids, peptides, and analogues	C28H34N8O4	[M + H3N + H]+	564.3047	564.3019	-4.8582	11.7575	0.86	Root
4-{[(1-methylethyl)carbamoyl]amino}-1-[(1-methyl-1H-pyrrol-2-yl)carbonyl]prolyl-O-methyltyrosinamide	Organic acids and derivatives	Carboxylic acids and derivatives	Amino acids, peptides, and analogues	C25H34N6O5	[M + Na]+	521.2488	521.2522	6.3856	19.7843	0.86	Leaf Root
L-isoleucyl-L-leucyl-L-valyl-L-tyrosine	Organic acids and derivatives	Carboxylic acids and derivatives	Carboxylic acid derivatives	C26H42N4O6	[M + Na]+	529.3002	529.3026	4.5326	24.668	0.86	Stem Leaf Root
2-[6-amino-8-[2-(1H-indol-3-ylmethylidene)hydrazinyl]purin-9-yl]-5-(hydroxymethyl)oxolane-3,4-diol	Organic oxygen compounds	Organooxygen compounds	Carbohydrates and carbohydrate conjugates	C19H20N8O4	[M + H]+	425.168	425.1658	-5.0948	22.255	0.86	Root
1,3-bis[(3-methylbutanoyl)oxy]propan-2-yl 13-methyltetradecanoate	Lipids and lipid-like molecules	Glycerolipids	Triradylglycerols	C28H52O6	[M + Na]+	507.3662	507.3653	-1.6081	20.6454	0.85	Stem Leaf Root
Ala-Gly-Leu-Leu-Gly-Gly	Organic acids and derivatives	Carboxylic acids and derivatives	Amino acids, peptides, and analogues	C21H38N6O7	[M + H]+	487.2874	487.2838	-7.4617	24.8763	0.85	Leaf Root
2''-amino-17'-(hydroxymethyl)-5''-{[2-(hydroxymethyl)-5-[(methylamino)methyl]phenyl]methyl}-18'-(3-hydroxypropyl)-2'-methyl-5'',6''-dihydro-1''H-dispiro[cyclohexane-1,7'-[5,6]dithiatetracyclo[10.6.2.0^{1,13}.0^{2,1}?]icosane-8',4''-pyrimidin]-12'-en-20'-one	Lipids and lipid-like molecules	Steroids and steroid derivatives	Unassigned	C41H62N4O4S2	[M + H]+	739.4284	739.4247	-5.0531	18.4893	0.85	Stem Fruit Leaf Root
2-[[(2S)-2-[[(2S)-2-[[(2S)-2-[[2-hydroxy-3-(methylamino)decanoyl]amino]-3-methylbutanoyl]-methylamino]-3-methylbutanoyl]-methylamino]-3-(4-	Organic acids and derivatives	Carboxylic acids and derivatives	Amino acids, peptides, and analogues	C41H63N5O9	[M + Na]+	792.4523	792.4473	-6.42	18.3326	0.85	Fruit Leaf Root

hydroxyphenyl)propanoyl]amino]-3-(4-hydroxyphenyl)propanoic acid											
N(1),N(8)-bis(caffeoyl)spermidine	Organic acids and derivatives	Carboxylic acids and derivatives	Amino acids, peptides, and analogues	C25H31N3O6	[M + H]+	470.2285	470.2269	-3.4203	11.0801	0.84	Stem Fruit Leaf
(2S)-N-[(2S,3S)-1-[[(2S)-1-[[(2S)-5-amino-1,5-dioxopentan-2-yl]amino]-1-oxopropan-2-yl]amino]-3-methyl-1-oxopentan-2-yl]-1-[(2S)-2-[[(2S)-2-aminopropanoyl]amino]-3-methylbutanoyl]pyrrolidine-2-carboxamide	Organic acids and derivatives	Carboxylic acids and derivatives	Amino acids, peptides, and analogues	C27H47N7O7	[M + Na]+	604.3435	604.3441	1.1301	20.4649	0.84	Fruit Leaf Root
Prostaglandin E2-biotinamide	Organoheterocyclic compounds	Biotin and derivatives	Unassigned	C35H58N4O6S	[M + H]+	663.4149	663.4132	-2.5971	27.8753	0.84	Leaf
Impulsin	Lipids and lipid-like molecules	Fatty Acyls	Fatty amides	C18H37NO2	[M + Na]+	322.2722	322.2729	2.1813	27.1344	0.84	Stem Leaf
Dusquetide	Lipids and lipid-like molecules	Steroids and steroid derivatives	Unassigned	C25H47N9O5	[M + Na]+	576.3598	576.3645	8.1299	17.0995	0.83	Fruit
2,2,6b,9,9,12a,14a-heptamethyl-10,11-dipropanoyl-docosahydropicene-4a-carboxylic acid	Lipids and lipid-like molecules	Prenol lipids	Triterpenoids	C36H58O4	[M + Na]+	577.4233	577.4236	0.5846	17.3742	0.83	Root
3beta-[4-O-(3-O-Methyl-6-deoxy-beta-D-allopyranosyl)-2,6-dideoxy-beta-D-arabino-hexopyranosyloxy]-8,11,12,14-tetrahydroxypregna-5-ene-20-one	Organic oxygen compounds	Organooxygen compounds	Carbohydrates and carbohydrate conjugates	C34H54O13	[M + Na]+	693.3462	693.3473	1.5422	12.9701	0.83	Stem Fruit Leaf Root
Cyclo(grgdspa)	Organic acids and derivatives	Carboxylic acids and derivatives	Amino acids, peptides, and analogues	C24H38N10O12	[M + Na]+	681.2568	681.2554	-2.1166	16.8498	0.83	Stem Fruit Leaf Root

[2-(2,5-Dioxo-hexahydro-imidazo[4,5-d]imidazol-1-yl)-acetylamino]-acetic acid	Organic oxygen compounds	Organooxygen compounds	Carbohydrates and carbohydrate conjugates	C8H11N5O5	[M + H3N + H]+	275.1104	275.1119	5.3311	13.0406	0.82	Root
Thr-Val-Glu-Glu-Val-Asp	Organic acids and derivatives	Carboxylic acids and derivatives	Amino acids, peptides, and analogues	C28H46N6O14	[M + K]+	729.2709	729.2758	6.7485	16.0209	0.81	Stem Leaf Root
[(6R,8Z,10R,11R,12S,13S,14S,15R,17R,18E,21S)-4-ethoxy-2,6,14,28-tetrahydroxy-10-methoxy-3,11,13,15,17,21-hexamethyl-25-[[(4-methylpiperazin-1-yl)amino]methyl]-16,22-dioxo-7-oxa-23-azatricyclo[22.3.1.05,27]octacosa-1(27),2,4,8,18,24(28),25-heptaen-12-yl] acetate	Organic acids and derivatives	Carboxylic acids and derivatives	Amino acids, peptides, and analogues	C43H64N4O11	[M + H]+	813.4644	813.4662	2.2628	26.8255	0.81	Leaf
[(2S)-2-tridecanoyloxy-3-[(2R,3S,4S,5R,6S)-3,4,5-trihydroxy-6-(hydroxymethyl)oxan-2-yl]oxypropyl] tetradecanoate	Organic oxygen compounds	Organooxygen compounds	Carbohydrates and carbohydrate conjugates	C36H68O10	[M + H]+	661.4884	661.4879	-0.8635	20.6653	0.81	Stem Leaf Root
N,N''-Bis(1-cyclohexyl-5-allyl-5-propylidenebarbituryl)diaminoguanidine	Organic acids and derivatives	Carboxylic acids and derivatives	Amino acids, peptides, and analogues	C33H47N9O6	[M + H]+	666.3721	666.3725	0.5262	24.5585	0.8	Leaf
Glyoxyloyl carbocysteine	Organic acids and derivatives	Carboxylic acids and derivatives	Carboxylic acids	C7H9NO6S	[M + H3N + H]+	253.0494	253.0519	9.9074	21.0057	0.8	Stem Leaf Root
[6-(hexadec-7-enoyloxy)-3,4,5-trihydroxyoxan-2-yl]methyl octadecanoate	Organic acids and derivatives	Carboxylic acids and derivatives	Amino acids, peptides, and analogues	C33H50N8O7	[M + K]+	709.344	709.3433	-0.8755	12.5007	0.8	Fruit Leaf Root
5-(3-ethoxy-4-hydroxyphenyl)-3-hydroxy-1-(2-methoxyethyl)-4-{[3-methyl-4-(methylethoxy)phenyl]carbonyl}-3-pyrrolin-2-one	Organic acids and derivatives	Carboxylic acids and derivatives	Amino acids, peptides, and analogues	C26H31NO7	[M + Na]+	492.1998	492.2025	5.4188	19.0439	0.79	Stem Leaf Root

5-[[4-[5-[[4-[5-[acetyl(hydroxy)amino]pentylamino]-4-oxobutanoyl]-hydroxyamino]pentylamino]-4-oxobutanoyl]-hydroxyamino]pentanoic acid	Organic oxygen compounds	Organooxygen compounds	Carbohydrates and carbohydrate conjugates	C25H45N5O10	[M + H3N + H]+	593.351	593.3552	7.1221	19.9038	0.79	Leaf Root
Ethyl 4-sulfonamidonaphthyl sulfone	Organic oxygen compounds	Organooxygen compounds	Carbohydrates and carbohydrate conjugates	C12H13NO4S2	[M + H3N + H]+	317.063	317.0645	4.8779	11.9577	0.79	Stem Leaf
(2S)-N-[(2S)-1-[[(2S)-1-[[(2S)-6-amino-1-oxohexan-2-yl]amino]-3-(4-hydroxyphenyl)-1-oxopropan-2-yl]amino]-3-methyl-1-oxobutan-2-yl]-2-[[(2S)-2-aminopropanoyl]amino]-4-methylpentanamide	Organic acids and derivatives	Peptidomimetics	Depsipeptides	C29H48N6O6	[M + H]+	577.3707	577.3706	-0.3068	19.421	0.79	Stem Fruit Leaf Root
[(4R,9R,10R,14R)-14-hydroxy-5,5,9-trimethyl-14-tetracyclo[11.2.1.01,10.04,9]hexadecanyl]methyl (4S,6R,9S,10R)-6-acetyloxy-5,5,9-trimethyltetracyclo[11.2.1.01,10.04,9]hexadecane-14-carboxylate	Lipids and lipid-like molecules	Glycerolipids	Diradylglycerols	C42H66O5	[M + Na]+	673.4808	673.4814	0.8734	33.8691	0.79	Stem Fruit Root
Ala-Arg-Thr-Nle(tBu)-Ala-al	Organic acids and derivatives	Carboxylic acids and derivatives	Amino acids, peptides, and analogues	C26H50N8O6	[M + Na]+	593.3751	593.3696	-9.2341	22.2828	0.79	Root
[(2S,3R,4S,5S,6R)-3,4,5-trihydroxy-6-(hydroxymethyl)oxan-2-yl] (1S,3S,4S,6S,7S,11S,12S,14S,15R,16R)-15-[(2S,5S)-2,5-dihydroxy-5-(hydroxymethyl)-6-methylheptan-2-yl]-4,6,14-trihydroxy-7,12,16-trimethylpentacyclo[9.7.0.01,3.03,8.012,16]octadecane-7-carboxylate	Lipids and lipid-like molecules	Steroids and steroid derivatives	Bile acids, alcohols and derivatives	C37H62O13	[M + Na]+	737.4088	737.4127	5.2837	16.9392	0.78	Root

4,4''-(4,4''-Bipyridine-1,1''-diyl)bis(2,6-diaminopyrimidine 1-oxide)	Organic acids and derivatives	Carboxylic acids and derivatives	Amino acids, peptides, and analogues	C18H28N10O2	[M + H]+	417.2469	417.2473	1.0362	13.453	0.78	Leaf Root
Ala-Ser-Leu-Ser(PO3H2)-Ala-Pro-Gly-al	Organic acids and derivatives	Carboxylic acids and derivatives	Amino acids, peptides, and analogues	C25H44N7O12P	[M + Na]+	688.2683	688.2648	-5.1311	15.9686	0.78	Stem Leaf Root
N-({2-[4-(isobutyrylamino)benzoyl]hydrazino}carbonothioyl)-2-thiophenecarboxamide	Benzenoids	Benzene and substituted derivatives	Benzenesulfonamides	C17H18N4O3S2	[M + K]+	429.0457	429.0436	-4.9157	20.0075	0.78	Leaf
3''-Nitro-4''-(lauryloxy)biphenyl-4-carboxylic aid	Benzenoids	Benzene and substituted derivatives	Phenoxy compounds	C31H45NO5	[M + Na]+	534.3195	534.3191	-0.7541	24.9303	0.77	Stem Leaf Root
Decyl-|A-D-maltoside	Organic oxygen compounds	Organooxygen compounds	Carbohydrates and carbohydrate conjugates	C22H42O11	[M + H]+	483.2799	483.2756	-8.8566	25.81	0.77	Leaf Root
3-({6-[2-amino-3-(methylazaniumyl)propoxy]-3,4-dihydroxy-5-{5,7,11,19-tetraoxapentacyclo[10.8.0.0²,¹?.0?,?.0¹³,¹?]icosa-2(10),3,8,13,15,17-hexaen-15-ylperoxy}oxan-2-yl}methoxy)-3-oxopropanoate	Organic oxygen compounds	Organooxygen compounds	Carbohydrates and carbohydrate conjugates	C29H34N2O14	[M + Na]+	657.1908	657.1931	3.5241	17.016	0.77	Leaf
N-[(Benzyloxy)carbonyl]-L-Leucyl-N-[(3s)-5-Methyl-1-(Methylsulfonyl)hexan-3-Yl]-L-Leucinamide	Organic acids and derivatives	Carboxylic acids and derivatives	Amino acids, peptides, and analogues	C28H47N3O7S	[M + H]+	570.3207	570.3163	-7.7507	16.1762	0.76	Leaf Root
6-[2,5-dihydroxy-7-methoxy-1-(3-methylbut-2-enyl)-9,10-dihydrophenanthren-3-yl]-7-methoxy-1-(3-methylbut-2-enyl)-9,10-dihydrophenanthrene-2,5-diol	Benzenoids	Benzene and substituted derivatives	Unassigned	C40H42O6	[M + K]+	657.2618	657.2653	5.2965	21.0075	0.76	Leaf Root
1H-Indazole-3-carboxamide, 1-(1-methylethyl)-N-(2-(4-((tricyclo(3.3.1.13,7)dec-1-	Organic acids and derivatives	Carboxylic acids and derivatives	Amino acids, peptides, and analogues	C29H41N5O2	[M + H3N + H]+	509.3604	509.3637	6.4224	18.7357	0.76	Leaf

ylcarbonyl)amino)-1-piperidinyl)ethyl)-											
(1R,4E,6S,7R,8S,10R,11R,12S,14R,15S,16R,18E,20E,22R,25S,29S,30S,32S,33S,34S)-22-ethyl-7,11,14,15-tetrahydroxy-30-[(2R)-2-hydroxypropyl]-6,8,10,12,14,16,29,33,34-nonamethyl-2,31-dioxa-26-oxoniatetracyclo[23.8.1.026,32.027,32]tetratriaconta-4,18,20-triene-3,9,13-trione	Lipids and lipid-like molecules	Sphingolipids	Ceramides	C45H72O11	[M + H]+	789.5147	789.5149	0.3138	28.7872	0.76	Leaf
Gly-Asn-Asn-Ser-Tyr-Ser	Organic acids and derivatives	Carboxylic acids and derivatives	Amino acids, peptides, and analogues	C25H36N8O12	[M + H]+	641.2525	641.2506	-2.9692	15.4567	0.75	Stem Fruit Leaf Root
1-Palmitoyl-2-myristoyl-3-myristoleoyl-glycerol	Lipids and lipid-like molecules	Glycerolipids	Triradylcglycerols	C47H88O6	[M + Na]+	771.6479	771.6448	-3.9883	29.4395	0.75	Leaf
16-benzyl-8,14,17-trioxo-6-(5-oxopyrrolidine-2-carbonyl)-2-oxa-6,9,15,18-tetraazatetracyclo[19.2.2.0^{3},?.0?,^{13}]pentacosa-1(23),21,24-triene-19-carboxylic acid	Organic acids and derivatives	Carboxylic acids and derivatives	Amino acids, peptides, and analogues	C33H37N5O8	[M + Na]+	654.254	654.2545	0.8161	17.0611	0.75	Stem Leaf Root
2,3,4,5-tetrahydroxy-6-(hydroxymethyl)cyclohexyl tetracosanoate	Lipids and lipid-like molecules	Prenol lipids	Tetraterpenoids	C31H60O7	[M + Na]+	567.4237	567.4201	-6.3295	29.1768	0.74	Leaf
Solphenazine F	Organic oxygen compounds	Organooxygen compounds	Carbohydrates and carbohydrate conjugates	C25H30N2O10	[M + Na]+	541.1798	541.1785	-2.5104	14.1364	0.74	Root
L-Prolinamide, N-(1-oxo-2-propylpentyl)-L-alpha-aspartyl-N-(4-((aminoiminomethyl)amino)-1-formylbutyl)-, (S)-	Organic acids and derivatives	Carboxylic acids and derivatives	Amino acids, peptides, and analogues	C23H40N6O6	[M + H]+	497.3081	497.3084	0.6254	25.5829	0.74	Stem Fruit Leaf Root
2-({bicyclo[3.1.1]heptan-2-ylidene}methoxy)-6-{[(3,4,5-trihydroxyoxan-2-yl)oxy]methyl}oxane-3,4,5-triol	Organic oxygen compounds	Organooxygen compounds	Carbohydrates and carbohydrate conjugates	C19H30O10	[M + H]+	419.1911	419.1888	-5.549	14.9155	0.74	Stem Leaf

3,7-O-diacetyl-5,14-O-dibenzoyl-13,17-oxypremyrsinol	Organoheterocyclic compounds	Naphthopyrans	Unassigned	C38H44O10	[M + H]+	661.3006	661.3025	2.8534	29.6017	0.74	Leaf
(3r,3as,4r,6ar)-4-(2-Methoxyethoxy)hexahydrofuro[2,3-B]furan-3-Yl {(2s,3r)-3-Hydroxy-4-[{[(2z)-2-(Methylimino)-2,3-Dihydro-1,3-Benzoxazol-6-Yl]sulfonyl}(2-Methylpropyl)amino]-1-Phenylbutan-2-Yl}carbamate	Organic acids and derivatives	Carboxylic acids and derivatives	Amino acids, peptides, and analogues	C32H44N4O10S	[M + H]+	677.285	677.2813	-5.4887	16.7803	0.73	Stem Leaf
Vicibactin-7101	Lipids and lipid-like molecules	Glycerolipids	Triradylcglycerols	C27H48N6O12	[M + H]+	649.3402	649.3458	8.5582	24.5585	0.73	Leaf
Dibekacin 2''-phosphate	Organic acids and derivatives	Carboxylic acids and derivatives	Amino acids, peptides, and analogues	C18H38N5O11P	[M + Na]+	554.2203	554.2202	-0.2334	14.2967	0.72	Fruit Leaf
Benzyl-dodecyl-bis(2-hydroxypropyl)azanium	Lipids and lipid-like molecules	Fatty Acyls	Fatty amides	C25H45NO2	[M + Na]+	414.3348	414.3354	1.4494	13.2563	0.72	Fruit Root
Oligomycin	Organic oxygen compounds	Organooxygen compounds	Carbohydrates and carbohydrate conjugates	C45H74O11	[M + Na]+	813.5129	813.5114	-1.7864	29.326	0.72	Leaf
[4-[(1-hydroxy-2-oxoazepan-3-yl)amino]-4-oxobutan-2-yl] 2-[[2-(2,3-dihydroxyphenyl)-4-methyl-5H-1,3-oxazole-4-carbonyl]amino]-6-[hydroxy-[(Z)-tetradec-5-enoyl]amino]hexanoate	Organic acids and derivatives	Carboxylic acids and derivatives	Amino acids, peptides, and analogues	C41H63N5O11	[M + Na]+	824.4422	824.4423	0.0999	14.2684	0.72	Fruit Root
dapt	Benzenoids	Benzene and substituted derivatives	Halobenzenes	C23H26F2N2O4	[M + Na]+	455.1758	455.1784	5.7047	15.2046	0.72	Fruit Leaf Root
Lexamine S 13	Organic acids and derivatives	Carboxylic acids and derivatives	Amino acids, peptides, and analogues	C23H48N2O	[M + H]+	369.3839	369.3831	-2.0471	29.8801	0.71	Leaf Root

[2-[2-[4,4-bis(hydroxymethyl)-5H-1,3-oxazol-2-yl]icos-4-enyl]-4-(hydroxymethyl)-5H-1,3-oxazol-4-yl]methanol	Benzenoids	Unassigned	Unassigned	C30H54N2O6	[M + Na]+	561.388	561.3853	-4.7629	27.6624	0.71	Stem Leaf Root
Hexadecaphytosphingosine	Organic nitrogen compounds	Organonitrogen compounds	Amines	C16H35NO3	[M + Na]+	312.2515	312.253	4.8324	26.9673	0.71	Stem Leaf Root
Proferrioxamine G1t	Organic acids and derivatives	Carboxylic acids and derivatives	Amino acids, peptides, and analogues	C23H46N6O7	[M + K]+	557.3065	557.3074	1.6312	25.8366	0.71	Stem Leaf Root
1-(9Z-tetradecenoyl)-glycero-3-phospho-(1''-myo-inositol)	Organic oxygen compounds	Organooxygen compounds	Carbohydrates and carbohydrate conjugates	C23H43O12P	[M + Na]+	565.239	565.2343	-8.3104	21.6444	0.71	Leaf Root
(2S)-2,6-bis[[(3R)-3-isocyanododecanoyl]amino]hexanoic acid	Organic acids and derivatives	Carboxylic acids and derivatives	Amino acids, peptides, and analogues	C32H56N4O4	[M + Na]+	583.4199	583.4157	-7.1633	29.4395	0.71	Leaf
6-[(2,4-dimethylpentan-3-yl)oxy]-3,4,5-trihydroxyoxane-2-carboxylic acid	Organic oxygen compounds	Organooxygen compounds	Carbohydrates and carbohydrate conjugates	C13H24O7	[M + H]+	293.1594	293.1606	4.0844	14.6466	0.71	Root
2-(2-octadec-9-enoxyethoxy)ethanol	Organic nitrogen compounds	Organonitrogen compounds	Amines	C22H44O3	[M + H3N + H]+	374.3634	374.3631	-0.7433	29.2847	0.71	Fruit Leaf
Dimethylaminopropyl palmitamide	Organic acids and derivatives	Carboxylic acids and derivatives	Amino acids, peptides, and analogues	C21H44N2O	[M + H]+	341.3526	341.3526	-0.0209	27.8318	0.7	Root
2-(2,2-dicyclopentylacetyl)oxyethyl-diethyl-octylazanium	Lipids and lipid-like molecules	Steroids and steroid derivatives	Steroidal alkaloids	C26H49NO2	[M + Na]+	430.3661	430.362	-9.4744	13.1038	0.7	Root
Ala-Gly-Tyr-Ala-Asn-Ala-Ala-al	Organic acids and derivatives	Carboxylic acids and derivatives	Amino acids, peptides, and analogues	C27H40N8O9	[M + Na]+	643.2816	643.2781	-5.4952	22.7392	0.7	Leaf
[2-({4-[(2-amino-4-oxo-4,7-dihydro-3H-pyrrolo[2,3-d]pyrimidin-5-yl)methyl]benzene-1-	Organic acids and derivatives	Carboxylic acids and derivatives	Amino acids, peptides, and analogues	C23H21N5O5	[M + H3N + H]+	465.1886	465.1908	4.5431	14.9388	0.7	Leaf

carbonyl}amino)-4-methoxyphenyl]acetic acid											
[(2R,3S,4S,5R,6S)-3,4,5-trihydroxy-6-[(1S,2R)-2-hydroxy-2-(2-hydroxy-2-methylpropyl)-4,4,6,6-tetramethyl-3,5-dioxocyclohexyl]oxyoxan-2-yl]methyl 3,4,5-trihydroxybenzoate	Organic acids and derivatives	Carboxylic acids and derivatives	Amino acids, peptides, and analogues	C27H38O14	[M + Na]+	609.2159	609.2177	2.9512	11.7166	0.7	Leaf Root
Itraconzole	Organic acids and derivatives	Carboxylic acids and derivatives	Amino acids, peptides, and analogues	C35H38F2N8O4	[M + K]+	711.2621	711.2645	3.3028	16.0045	0.69	Stem Leaf Root
33,34-dihydroxy-5,22-diazapentacyclo[16.16.1.0^{1,22}.0^{2,2}?.0?,^{3}?]pentatriaconta-10,26,29,31-tetraene-18-carbaldehyde	Organic acids and derivatives	Carboxylic acids and derivatives	Amino acids, peptides, and analogues	C34H52N2O3	[M + H]+	537.405	537.4089	7.1934	29.7347	0.69	Leaf
N-[(3R,6S,9S,12R,14Z,16E)-9-[3-(diaminomethylideneamino)propyl]-6-ethyl-12-methyl-2,5,8,11-tetraoxo-3-phenyl-1,4,7,10-tetrazacyclooctadeca-14,16,18-trien-12-yl]-2-methylpropanamide	Organic acids and derivatives	Carboxylic acids and derivatives	Amino acids, peptides, and analogues	C31H44N8O5	[M + Na]+	631.3332	631.337	5.9055	24.5585	0.69	Leaf
3-hydroxy-4-[17-(6-hydroxy-4,4,7a-trimethyl-2,4,5,6,7,7a-hexahydro-1-benzofuran-2-yl)-4,8,13-trimethyloctadeca-2,4,6,8,10,12,14,16-octaen-1-ylidene]-3,5,5-trimethylcyclohexyl acetate	Lipids and lipid-like molecules	Prenol lipids	Tetraterpenoids	C43H60O5	[M + H3N + H]+	674.4784	674.4812	4.0646	19.4464	0.69	Stem Leaf
Phenyl beta-D-glucopyranoside	Organic oxygen compounds	Organooxygen compounds	Alcohols and polyols	C12H16O6	[M + H]+	257.1019	257.1021	0.6914	15.9855	0.69	Fruit Leaf Root
5-methyltetrahydrofolyl polyglutamate	Organic acids and derivatives	Carboxylic acids and derivatives	Amino acids, peptides, and analogues	C30H39N9O12	[M + H]+	718.279	718.2816	3.5354	16.2858	0.69	Stem Fruit Leaf Root

7-[(2S)-2,3-dihydroxypropyl]-3H-purine-2,6-dione	Organoheterocyclic compounds	Azoles	Unassigned	C8H10N4O4	[M + Na]+	249.06	249.0614	5.6095	22.2335	0.69	Leaf
K98 Polysaccharide	Organic oxygen compounds	Organooxygen compounds	Carbohydrates and carbohydrate conjugates	C24H40O19	[M + H]+	633.2236	633.2232	-0.6724	23.5292	0.69	Root
Terpendole E	Lipids and lipid-like molecules	Unassigned	Unassigned	C28H39NO3	[M + H]+	438.3002	438.3013	2.5323	16.5111	0.69	Fruit Leaf Root
6-{[4-(dimethylamino)-3-hydroxy-6-methyloxan-2-yl]oxy}-16-ethyl-4,10-dihydroxy-7-(2-hydroxyethyl)-5,9,13,15-tetramethyl-1-oxacyclohexadeca-11,13-dien-2-one	Organic oxygen compounds	Organooxygen compounds	Carbohydrates and carbohydrate conjugates	C31H55NO8	[M + Na]+	592.3825	592.384	2.4864	14.4001	0.68	Fruit Leaf Root
(3alpha,5beta,7alpha,8xi,12alpha)-24-(4-Benzylpiperazin-1-yl)-3,7,12-trihydroxycholan-24-one	Organic acids and derivatives	Carboxylic acids and derivatives	Amino acids, peptides, and analogues	C35H54N2O4	[M + H3N + H]+	584.4427	584.4453	4.3403	30.18	0.68	Leaf
[3,8,14,17-tetrahydroxy-10,13-dimethyl-17-[1-(3-methylbutanoyloxy)ethyl]-1,2,3,4,5,6,7,9,11,12,15,16-dodecahydrocyclopenta[a]phenanthren-12-yl] 3-methylbutanoate	Organic oxygen compounds	Organooxygen compounds	Carbohydrates and carbohydrate conjugates	C31H52O8	[M + Na]+	575.356	575.358	3.573	22.4507	0.68	Root
Tris(diethylaminomethyl)phosphine oxide	Lipids and lipid-like molecules	Fatty Acyls	Unassigned	C15H36N3OP	[M + Na]+	328.2494	328.2481	-3.9507	25.3301	0.68	Stem Fruit Leaf Root
[(2S)-1-decanoyloxy-3-[(2R,3R,4S,5R,6R)-3,4,5-trihydroxy-6-(hydroxymethyl)oxan-2-yl]oxypropan-2-yl] hexadecanoate	Lipids and lipid-like molecules	Glycerolipids	Diradylglycerols	C35H66O10	[M + Na]+	669.4554	669.4501	-7.8589	33.8738	0.67	Leaf
2-[(1S,4S,5S)-4-[(benzylcarbamoylamino)methyl]-2-methyl-5-propan-2-ylcyclohex-2-en-1-yl]-N-(2-morpholin-4-ylethyl)acetamide	Organic acids and derivatives	Carboxylic acids and derivatives	Amino acids, peptides, and analogues	C27H42N4O3	[M + K]+	509.2894	509.2923	5.7169	17.3838	0.67	Leaf Root

1-(4-fluorophenyl)-4-{methyl[2-(1,3,4,9-tetrahydro-2H-beta-carbolin-2-yl)ethyl]amino}butan-1-one	Benzenoids	Benzene and substituted derivatives	Halobenzenes	C24H28FN3O	[M + H]+	394.2288	394.2281	-1.7815	24.758	0.67	Leaf Root
Roseolide A	Lipids and lipid-like molecules	Prenol lipids	Unassigned	C42H60O10	[M + Na]+	747.4084	747.408	-0.597	25.1514	0.67	Stem Fruit Leaf Root
6-{[3-(dimethylamino)-2-hydroxy-5-methylcyclohexyl]oxy}-16-ethyl-4-hydroxy-15-(hydroxymethyl)-5,7,9,13-tetramethyl-1-oxacyclohexadec-11-ene-2,10-dione	Lipids and lipid-like molecules	Steroids and steroid derivatives	Steroidal glycosides	C31H55NO7	[M + Na]+	576.3876	576.3871	-0.9499	14.3995	0.67	Fruit Root
3,3'-Methylenebis(4-hydroxy-6-methyl-2H-pyran-2-one)	Organoheterocyclic compounds	Pyrans	Pyranones and derivatives	C13H12O6	[M + Na]+	287.0532	287.055	6.3733	13.663	0.67	Stem Leaf
1-palmitoyl-3-alpha-D-galactosyl-sn-glycerol	Organic oxygen compounds	Organooxygen compounds	Carbohydrates and carbohydrate conjugates	C25H48O9	[M + Na]+	515.3196	515.3216	3.7906	26.2643	0.67	Stem Leaf Root
Burixafor	Organoheterocyclic compounds	Azoles	Unassigned	C27H51N8O3P	[M + Na]+	589.3719	589.3729	1.6553	23.0873	0.67	Root
2-[(Carboxymethyl)thio]-5-nitrobenzoic acid	Benzenoids	Benzene and substituted derivatives	Benzoic acids and derivatives	C9H7NO6S	[M + H3N + H]+	275.0338	275.0332	-2.0083	20.8442	0.66	Stem Leaf Root
methyl 2-[[2-[(17-hydroxy-10,13,17-trimethyl-7,8,9,11,12,14,15,16-octahydro-6H-cyclopenta[a]phenanthren-3-ylidene)amino]oxyacetyl]amino]-3-(1H-indol-3-yl)propanoate	Organic acids and derivatives	Carboxylic acids and derivatives	Amino acids, peptides, and analogues	C34H43N3O5	[M + H3N + H]+	591.3546	591.3494	-8.9292	19.4784	0.66	Fruit Leaf Root
methyl 1-[3-[(2S,5aS,8aR)-1-methyl-6-[(1-methylindol-3-yl)methyl]-5-oxo-3,4,5a,7,8,8a-hexahydro-2H-pyrrolo[3,2-e][1,4]diazepin-2-	Lipids and lipid-like molecules	Steroids and steroid derivatives	Unassigned	C28H39N5O4	[M + Na]+	532.29	532.2877	-4.2077	16.6766	0.66	Leaf Root

yl]propanoyl]piperidine-4-carboxylate											
Ala-Thr-Leu-Leu-Asp-Ala-al	Organic acids and derivatives	Carboxylic acids and derivatives	Amino acids, peptides, and analogues	C26H46N6O9	[M + H]+	587.3398	587.3425	4.4682	15.099	0.66	Fruit Root
1-[(3R,9R,10S)-9-[(dimethylamino)methyl]-12-[(2S)-1-hydroxypropan-2-yl]-3,10-dimethyl-13-oxo-2,8-dioxa-12-azabicyclo[12.4.0]octadeca-1(14),15,17-trien-16-yl]-3-(3,5-dimethyl-4-isoxazolyl)urea	Organic acids and derivatives	Carboxylic acids and derivatives	Amino acids, peptides, and analogues	C29H45N5O6	[M + H3N + H]+	577.3714	577.3677	-6.3191	17.3565	0.66	Root
N''-(2-{3-[(cyclohexyloxy)methyl]-4-[3-(cyclopentyloxy)-2-hydroxy-5-(3-oxooct-4-en-1-yl)phenyl]-6-methyl-2,3,4,4a,5,6,7,8-octahydronaphthalen-2-yl}ethyl)guanidine	Organic acids and derivatives	Carboxylic acids and derivatives	Amino acids, peptides, and analogues	C40H61N3O4	[M + K]+	686.4299	686.4274	-3.6294	14.6137	0.65	Root
S-(3Alpha,7Alpha,12Alpha-Trihydroxy-5Beta-Cholestan-26-Oyl)-4'-Phosphopantetheine	Organic acids and derivatives	Carboxylic acids and derivatives	Amino acids, peptides, and analogues	C38H67N2O11PS	[M + H]+	791.4275	791.4224	-6.4754	21.3521	0.65	Leaf Root
