# Supplementary material for: Metabolomic Analysis of Phytochemical Compounds from Agricultural Residues of Eggplant (Solanum melongena L.)
Source: Molecules. 2022 Oct 18;27(20):7013. doi: 10.3390/molecules27207013 (PMC9608517; doi:10.3390/molecules27207013)
Supplement: Supplementary file 1 [file molecules-27-07013-s001.zip › Supplementary_Material_Eggplant_.pdf]

SUPPORTING INFORMATION

Supplemental experimental methods

Metabolomics data processing**.** For the MZmine pipeline or approach 1, raw data files (scan format) were first converted to mzXML using MSconvert (ProteoWizard version 3.0.5471) [94]**.** Mass detection, chromatogram building and deconvolution (ADAP algorithm), isotopic assignment, feature alignment, and gap-filling (to detect features missed during the initial alignment) were performed in MZmine Version 2.53 [89]. Features detected in the mobile phase (water/acetonitrile 95:5 *v*/*v* with 0.1% formic) blanks were considered contaminants and removed from samples using the *peak list row filter* tool within MZmine version 2.53 and manual inspection. The list containing all MS^1 was exported as a .csv file. The overlapping pattern of the features among the plant's anatomical sites was determined by UpSet plotting using the UpSetR app version 1.4.0 (open source R package) [95–96]**.** For approach two, mzXML files were uploaded to the global natural products social molecular network (GNPS, https://gnps.ucsd.edu) to perform classical molecular networking and automated structural annotation (Metabolomics Standards Initiative [MSI] classification level 2) (against GNPS public spectral libraries) [91]**.** The parameters used within the GNPS were precursor ion and product ion mass tolerance of 0.02 Da. The molecular network was created using a minimum cosine score of 0.6 and a minimum of 4 peaks matched. The spectra in the network were searched against GNPS spectral libraries using a minimum cosine score of 0.6 and at least 4 matched peaks as filters. MS cluster option was selected. To expand the annotation of the metabolites not automatically retrieved by spectral matching, we employed advanced *in-silico* annotation tools to determine the metabolite's chemical classes (MSI, level 3) and molecular structures (MSI, level 3). Converted mzXML files were imported into SIRIUS software version 4.9.12 [97] for chemical formula [98]**,** molecular structure [99], and chemical class assignment [100]**.** To speed up running times, we selected only compounds <860 Da. Annotations with a COSMIC Score> 0.65 were only considered. Common contaminants (e.g., plasticizers, silanones) and halogen- and boron-containing metabolites were removed from the analysis. The overlapping pattern of the chemical classes among the plant's anatomical sites was determined by UpSet plotting using the ComplexUpSet package based on the UpSetR package in R [95–96]. Molecular structures were drawn using ChemDraw Professional version 16.0.1.4.
